# The central role of Sphingosine kinase 1 in the development of neuroendocrine prostate cancer (NEPC): A new targeted therapy of NEPC

**DOI:** 10.1002/ctm2.695

**Published:** 2022-02-20

**Authors:** Cheng‐Fan Lee, Yu‐An Chen, Elizabeth Hernandez, Rey‐Chen Pong, Shihong Ma, Mia Hofstad, Payal Kapur, Haiyen Zhau, Leland WK Chung, Chih‐Ho Lai, Ho Lin, Ming‐Shyue Lee, Ganesh V Raj, Jer‐Tsong Hsieh

**Affiliations:** ^1^ Department of Urology University of Texas Southwestern Medical Center Dallas Texas USA; ^2^ Department of Biochemistry and Molecular Biology College of Medicine National Taiwan University Taipei Taiwan; ^3^ Urology and Pathology University of Texas Southwestern Medical Center Dallas Texas USA; ^4^ Uro‐Oncology Research Department of Medicine Cedars‐Sinai Medical Center Los Angeles California USA; ^5^ Department of Microbiology and Immunology Graduate Institute of Biomedical Sciences College of Medicine Chang Gung University Taoyuan Taiwan; ^6^ Department of Life Sciences National Chung Hsing University Taichung Taiwan; ^7^ Department of Pharmacology University of Texas Southwestern Medical Center Dallas Texas USA

**Keywords:** neuroendocrine prostate cancer, Sphingosine kinase 1, targeted therapy, therapy and castration resistant prostate cancer

## Abstract

**Background:**

Neuroendocrine prostate cancer (NEPC) is often diagnosed as a sub‐type from the castration‐resistant prostate cancer (CRPC) recurred from the second generation of anti‐androgen treatment and is a rapidly progressive fatal disease. The molecular mechanisms underlying the trans‐differentiation from CRPC to NEPC are not fully characterized, which hampers the development of effective targeted therapy.

**Methods:**

Bioinformatic analyses were conducted to determine the clinical correlation of sphingosine kinase 1 (SphK1) in CRPC progression. To investigate the transcriptional regulation SphK1 and neuroendocrine (NE) transcription factor genes, both chromosome immunoprecipitation and luciferase reporter gene assays were performed. To demonstrate the role of SphK1 in NEPC development, neurosphere assay was carried out along with several biomarkers determined by quantitative PCR and western blot. Furthermore, in vivo NEPC xenograft models and patient‐derived xenograft (PDX) model were employed to determine the effect of SphK1 inhibitors and target validation.

**Results:**

Significant prevalence of SphK1 in NEPC development is observed from clinical datasets. SphK1 is transcriptionally repressed by androgen receptor‐RE1‐silencing transcription factor (REST) complex. Furthermore, sphingosine 1‐phosphate produced by SphK1 can modulate REST protein turnover via MAPK signaling pathway. Also, decreased REST protein levels enhance the expression of NE markers in CRPC, enabling the transition to NEPC. Finally, specific SphK1 inhibitors can effectively inhibit the growth of NEPC tumors and block the REST protein degradation in PDX.

**Conclusions:**

SphK1 plays a central role in NEPC development, which offers a new target for this lethal cancer using clinically approved SphK1 inhibitors.

## Graphical abstract

- Long‐term hormonal therapy‐elicited sphingosine kinase 1 (SphK1) gene transcription is modulated by androgen receptor (AR) and RE‐1 silencing transcriptional factor (REST) repressor complex.
- SphK1 produces sphingosine‐1‐phosphate (S1P) to promote trans‐differentiation of androgen‐dependent prostate cancer (ADPC) into neuroendocrine prostate cancer (NEPC) in an autocrine manner.
- The binding of S1P to its receptors (S1PRs) activate Erk1/2 to phosphorylate REST at serine 861/864 sites leading to proteasomal degradation, which unleashes transcriptional repression of neuronal transcriptional factors expression.
- FDA approved SphK1‐specific inhibitors (FTY720 or SKI‐II) can overcome Enzalutamide‐resistant CRPC tumor growth.

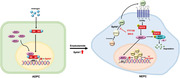

## 1 BACKGROUND

Prostate cancer (PCa) is an androgen‐dependent disease and androgen deprivation therapy (ADT) is considered the most effective regimen to treat metastatic disease. However, almost all patients eventually develop castration‐resistant PCa (CRPC) within 12 to 18 months of treatment with a median survival of 14 to 26 months, which is associated with the majority of mortality of this disease.^1^ Although new agents, such as anti‐androgen therapeutics (Enzalutamide or Abiraterone) or radiotherapy (Radium‐223) or immunotherapy (Sipuleucel‐T), have been introduced for these patients, CRPC inevitably acquires resistance known as therapy and castration‐resistant PCa (t‐CRPC) ^2^, ^3^ Clinical observations^4^, ^5^ indicate that t‐CRPC often exhibits distinct neuroendocrine (NE) phenotype with neuronal progenitor transcription factors (NETFs) expression and neuronal factors secretion in an endocrine fashion,^6^ which is initiated by lineage plasticity of PCa leading to NE differentiation (NED) during CRPC progression.^7^ Since NE PCa (NEPC) is resistant to ADT or radiotherapy,^8^, ^9^ unveiling the key molecular mechanism associated with NEPC progression could certainly lead to the development of new targeted therapeutics for NEPC.

The majority of PCa is adenocarcinoma (ADPC) that express androgen receptor (AR). In contrast, NEPC is characterized by a loss of AR expression, which attributes to ADT resistance. Also, the distinct expression of several NETFs (such as BRN2, EZH2, FOXA2 SOX2) and NE markers, such as chromogranin A (CgA) and synaptophysin (Syp), are associated with NEPC.^10^, ^11^, ^12^, ^13^ It is believed that NEPC cells are trans‐differentiated from ADPC. Recent studies unveil several intrinsic genetic drivers of NEPC such as loss of function of mutation in Rb and TP53 genes,^13^, ^14^ N‐MYC amplification,^15^ or overexpression of Aurora A kinase.^16^ On the other hand, exogenous factors, including cytokines and growth factors,^17^ are capable of inducing NE phenotypes in ADPC cell lines in vitro, suggesting an epigenetic regulation of NED.

The relationship between PCa development and lipid metabolism is well established.^18^ Many studies showed an increase in total cholesterol and triglycerides levels upon treatment of ADT;^19^, ^20^ these changes support the notion that AR regulates lipid metabolism. Thus, it is believed that PCa patients receiving long‐term (at least 12 months) ADT have a greater risk for metabolic syndrome compared with the men in the control groups.^21^, ^22^ However, the effect of lipid metabolism on NEPC progression is not well known. In this study, we report the promoting effect of sphingosine kinase 1 (SphK1) but not SphK2 on NEPC development, which could arise from either genetic alteration or transcriptional regulation mediated by the AR‐ RE1‐silencing transcriptional factor (REST) complex. We have demonstrated that SphK1, catalyzes sphingosine to sphingosine‐1 phosphate (S1P), which can promote NED of ADPC cells. Mechanistically, upon binding to its specific receptors, S1P can specifically activate the ERK signaling network, whose function is to accelerate the turnover of REST protein after phosphorylation leading to de‐repression of many NETF genes transcription. Our findings reveal a new molecular mechanism by which NEPC development can be regulated by a lipid metabolite and further support Sphk1 as a potent therapeutic target for eradicating NEPC.

## 2 MATERIALS AND METHODS

### 2.1 Cell models, neurosphere assay and gene transfection

LNCaP, C4‐2, C4‐2B, 22RV1, NCI‐H660 and PC3 were maintained in RPMI‐1640 (Sigma‐Aldrich) supplemented with 10% FBS (Gibco), 2 mM l‐glutamine (Sigma‐Aldrich), 1% Penicillin/Streptomycin (P/S) (Hyclone). VCAP cells were provided by Kenneth Pienta (Johns Hopkins University, Baltimore, MD, USA) and cultured in DMEM (Sigma‐Aldrich) supplemented with 10% FBS, 2 mM l‐glutamine and 1% P/S (Hyclone). IIB5 and IIG5 were single‐cell clones derived from ARCaP^23^ maintained in RPMI‐1640 with 10% FBS, 2 mM l‐glutamine and 1% v/v P/S. All cell lines were used within 10 passages and authenticated with the short tandem repeat (STR) profiling by Genomic Core in UT Southwestern Medical Center (UTSW) periodically. Mycoplasma testing was performed by MycoAlert kit (Lonza Walkersville) every quarter to ensure cells remained mycoplasma free.

Neurosphere assay^24^ was carried out by plating cells (200–300 cells) in ultra‐low plate containing Neurobasal medium (ThermoFisher Scientific), B27 Plus Supplement (ThermoFisher Scientific), 20 ng/mL basic fibroblast growth factor‐2 (ThermoFisher Scientific) and 20 ng/mL epidermal growth factor (ThermoFisher Scientific) for 10 days. The number of sphere (larger than 50 μm in diameter) imaged by microscope was counted to determine sphere‐forming ability.

For gene transfection, cells (2.5 × 10^5^) were seeded in a 60‐mm dish at 60–70% confluency then transfected with plasmid vectors containing cDNA, luciferase reporter gene promoter or small hairpin RNAs (shRNA) constructs obtained from the National RNAi Core Facility (Academia Sinica) using either Xfect (Clontech) or JetPEI (Q‐Bio Gene) transfection kit according to manufacturers’ protocol.

### 2.2 CRISPR/Cas9 gene knockout, small hairpin RNA knockdown and lentiviral particle preparation

Based on Feng Zhang's CRISPR guide design tool (http://crispr.mit.edu/), the guide RNAs for SphK1 gene knockout were designed: Ex2 or Ex3 (Supporting information Table S1) and subsequently cloned into lentiCRISPRv2 (Addgene). Small hairpin RNAs (shAR [TRCN0000314657]) for AR knockdown were obtained from the National RNAi Core Facility of Academia (Sinica, Taiwan). The PLKO vector was used as control.

For lentiviral particle preparation, Lenti‐X 293T cell line (TakaRa) was used for transfection according to the manufacturer's protocol. Cells were cultured with OPTI‐MEM serum‐free media and transfected with the mixture of lentiviral package plasmids (pCMV‐ΔR8.91 and pMD.G) then viral suspension was harvested 24 h afterwards. The supernatant was then filtrated through a 0.45μm Steri‐Flip filter (Millipore) and used for cell infection.

### 2.3 Site‐directed mutagenesis

The WT SphK1 tagged with FLAG at the carboxyl terminal was provided by Dr. Bink Wattenberg (James Graham Brown Cancer Center, KY, USA). The plasmid was used as the template to generate two SphK1 mutants (CA S225E and DN S225A) using site‐directed mutagenesis kit (Agilent); the oligonucleotides were used for SphK1 CA S225E or DN S225A (Supporting information Table S1).

### 2.4 Chromatin immunoprecipitation (ChIP), ChIP‐ reChIP and ChIP sequencing (ChIP‐seq)

ChIP assay was performed by using ChIP‐IT Express Enzymatic kit (Active Motif) according to the manufacturer's instructions. Briefly, cells were cross‐linked with 1% formaldehyde for 10 min, quenched with glycine followed by nuclear lysis. After isolating nuclear fractions, chromatin was enzymatically sheared into 200–100 bp. The sheared DNA was immunoprecipitated with ChIP‐grade antibodies for 16 h. After reversal of cross‐linking, DNA fragments were purified on spin columns (Active Motif) subjected to DNA‐seq. AR ChIP‐REST reChIP was performed by using Magna ChIP A/G kit (Millipore) according to the manufacturer's instructions. Briefly, after treatment, cells were cross‐linked with 1% formaldehyde for 10 min at room temperature and quenched with glycine followed by nuclear lysis. After isolating nuclear fractions, chromatin was sonicated and sheared into 200–100 bp with condition of amplitude: 30%, 15 s ON, 30 s OFF for 10 cycles. The sheared DNA was incubated with Magna‐conjugated AR (Cell Signaling) antibody for 16 h and then separated into two portions. One portion of precipitated complex, after reversal of cross‐linking, DNA fragments were purified on spin columns (Millipore) subjected to real‐time PCR. For REST reChIP, AR ChIP complex was washed with stripping buffer for 1 h and incubated with Magna‐conjugated REST antibody (Abclonal) for 16 h then samples were immunoprecipitated with Magna‐conjugated bead. After reversal of cross‐linking, purified DNA fragments with spin columns (Millipore) were followed by real‐time PCR. The data were normalized to IgG group. The primers used in this study are listed in Supporting information Table S1 and all the antibodies used in this study are listed in Supporting information Table S2.

### 2.5 Gene promoter construction and luciferase reporter assay

Cells (2 × 10^5^ per well) stably transfected with luciferase reporter plasmids were plated in six‐well plate and were transfected with REST S861/864A plasmid using Xfect (Clontech) for 16 h. Next day, all cells were serum starved for 2 h before S1P (2.5 μM) treatment for 4 h then subjected to luciferase reporter assay (Promega) determined by Monolight TD 20/20 luminometer (Turner Designs). The relative reporter gene activity was normalized with protein concentration. All transfection experiments were performed in triplicates.

### 2.6 RNA isolation and quantitative real‐time RT‐PCR (qRT‐PCR)

Total cellular RNA was extracted using Maxwell 16 LEV SimplyRNA Purification Kit (Promega) and 2 μg RNA was reversely transcribed into cDNA using iScript cDNA Synthesis Kit (Bio‐Rad). Real‐time PCR analysis was set up with SsoAdvanced Universal SYBR Green Supermix Kit (BioRad) and carried out in MyiQ thermal cycler (Bio‐Rad). All quantitative data of mRNA expression level were analyzed using Δ*Ct* (*Ct* value normalized to 18S RNA) and ΔΔ*Ct* (difference between the Δ*Ct* of control and experimental groups) values to obtain the fold change after normalizing with control group. The primers used in this study are listed in Supporting information Table S1.

### 2.7 Western blot analyses and immunoprecipitation (IP)

Cells were lysed in ice‐cold lysis buffer [150 mmol/L NaCl, 1% Triton X‐100, 0.5% sodium deoxycholate, 0.1% SDS, 50 mmol/L Tri (pH 8.0), protease inhibitor cocktail (Roche)] and cell debris were removed by centrifugation at 4°C for 10 min at 12,000 rpm. Equal amount of protein was subjected to electrophoresis on NuPAGE gels (Life Technologies) then transferred onto nitrocellulose membranes. Subsequently, membranes were blocked with 2% non‐fat dry milk w/v for 1 h and then incubated with primary antibodies (Supporting information Table S2). Appropriate secondary antibodies conjugated with horseradish peroxidase and enhanced chemiluminescence were used to detect target proteins. Results were visualized with ECL chemiluminescent detection system (Pierce ThermoScientific). For immunoprecipitation (IP), AR antibody was pre‐incubated with protein G Magna bead (Cell Signaling) for 1 h then 300 μg protein lysate was incubated with Magna‐conjugated AR for 16 h at 4°C and spun down. After washing three times, immunocomplex was eluted with buffer containing β‐mercaptoethanol then heat denatured before SDS‐PAGE. The relative protein expression level in each sample was normalized to actin.

### 2.8 Determination of S1P production

Cells (1 × 10^4^ cells per well) were seeded in 96‐well plate with regular culture medium overnight then replaced by 200 μL serum‐free RPMI medium for 24 h. The supernatant was collected after centrifugation and subjected to ELISA kit (MyBioSource, MBS069092) for determining S1P production. For the patient‐derived tumor explant (PDE) samples, 10 μg of the homogenized tissue samples were subjected to ELISA. Three independent experiments were performed for statistical calculation and presented as mean ± SD.

### 2.9 In vitro cytotoxicity assay

Cells (5000 cells/well) were seeded onto the 96‐well plate. After 24 h, fresh media containing different concentrations of FTY720 (Selleckchem), SKI‐II (Selleckchem) or Opaganib (Selleckchem) were incubated for 48 h. In vitro cytotoxicity was measured using. MTT (3‐(4,5‐Dimethylthiazol‐2‐yl) ‐2,5‐Diphenyltetrazolium Bromide, Sigma‐Aldrich) assay according to the manufacturer´s instructions. The relative number of viable cells was determined using a microtiter plate reader at OD_570_ nm. The experiment was repeated in triplicate and data were represented as mean ± SD.

### 2.10 Tissue microarray and immunohistochemistry (IHC)

Formalin‐fixed, paraffin‐embedded sections were de‐paraffinized, rehydrated and subjected to heat‐induced antigens retrieval (citrate buffer, pH 6.0) then incubated with appropriate primary antibody (Supporting information Table S2) and developed with 3, 3′‐diaminobenzidine chromogen followed by counterstaining with hematoxylin and eosin. The H‐score of immunohistochemistry (IHC) was calculated by percentage of cytosolic staining (0: 0–5%; 1: 5–25%; 2: 25–75%; 3: 75–100%) × staining intensity (0: no staining; 1: weak staining; 2: moderate staining; 3: strong staining).

### 2.11 Tumor xenografts

Three different NEPC (1 × 10^6^ cells/site) cells were injected subcutaneously into the flank of castrated male NOD‐SCID (6–8 weeks) mice. Once tumors became palpable, intraperitoneal injection of vehicle DMSO, FTY720 (15 mg/kg), SKI‐II (15 mg/kg) or Enzalutamide (20 mg/kg) was injected three times per week for 2 weeks, then tumor volume was determined by caliper and calculated (length × width × width/2). All animal work was approved by the Institutional Animal Care and Use Committee.

### 2.12 Patient‐derived tumor explants (PDEs)

The ex vivo explant culture was performed as previously described.^25^ Briefly, fresh PCa tissues were dissected into 1 mm^3^ cube and placed on a Gelatin sponge (Novartis) bathed in RPMI‐1640 media supplemented with 10% FBS, 100 units/mL P/S, 0.01 mg/mL hydrocortisone and 0.01 mg/mL insulin (Sigma‐Aldrich). Tissues were treated with FTY720 or SKI‐II (30 μM) for 48 h then were subjected to western blot analyses. The Institutional Review Board of UTSW approved the tissue procurement protocol for this study and written informed consent was obtained from all patients.

### 2.13 Bioinformatics and statistical analyses

RNA‐seq data were statistically analyzed based on the TCGA and CRPC database.^26^ PCa patient survival rates were analyzed based on NCBI's Gene Expression Omnibus (GEO). The Prism Statistics (GraphPad) was used for all statistical analysis. Statistical significance, *p *< .05 (*) and *p *< .01 (**), was analyzed by Student's *t*‐test. Data (mean ± SD) represented at least three independent experiments.

## 3 RESULTS

### 3.1 SphK1 is associated with NEPC development and confers Enzalutamide resistance of ADPC

**FIGURE 1:**
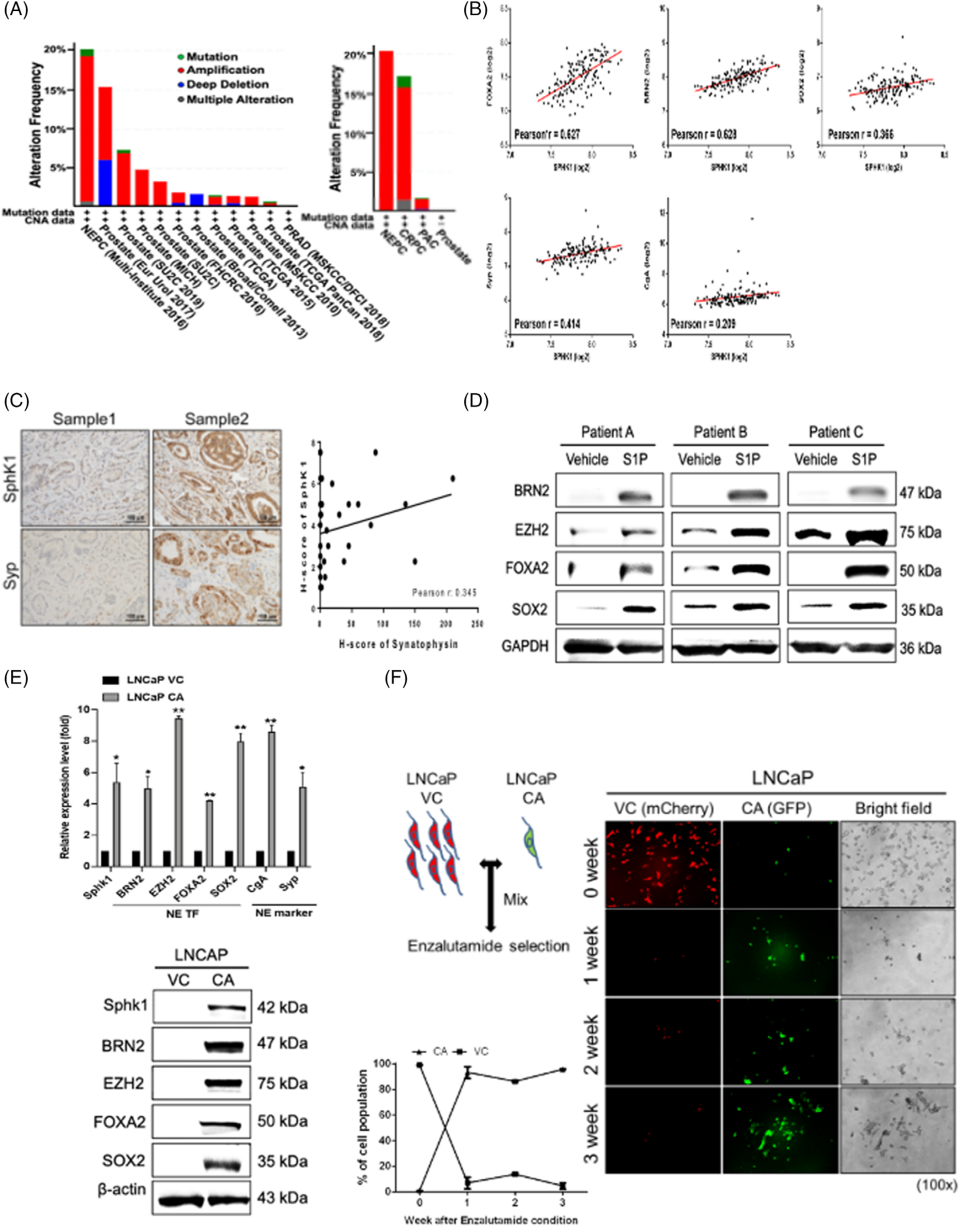
The association of Sphk1 expression with NEPC progression. (A) The frequency of SphK1 gene alterations (green: mutation; blue: deletion; red: amplification) in PCa patients (cBioPortal database). (B) The positive correlation of mRNA expression between SphK1 and NE‐related genes (BRN2, FOXA2, SOX2, CgA, Syp) in PCa patients (Betastasis Database). (C) The positive correlation of SphK1 and Syp expression using IHC on PCa TMA (n = 44). (D) Increased NETFs (BRN2 and FOXA2) and NE marker (Syp) protein expression in patient‐derived explants treated with 100 μM S1P for 24 h. (E) The induction of NETFs and NE markers in LNCaP cells expressing CA‐SphK1. (F) The stimulatory effect of CA‐SphK1 on the growth of LNCaP under androgen deprived condition (20 μM Enzalutamide). Left top panel: Scheme of co‐culture of VC cells (mCherry^+^) and CA‐SphK1 cells (GFP^+^) at ratio 100:1 incubated with 20 μM Enzalutamide for 3 weeks. Left bottom panel: Cell growth of two different LNCaP sub‐lines treated with Enzalutamide. Right panel: Images of two different LNCaP sub‐lines treated with Enzalutamide. *p < .05; **p < .01

Although PCa is a well‐characterized lipid‐rich tumor, the role of lipid metabolism or metabolite in PCa progression is not fully characterized. SphK, with two isoforms (SphK1 and SphK2), is a bioactive enzyme capable of converting sphingosine into S1P, which is a lipid mediator playing a major regulatory role in tumor cell growth, survival, invasion, angiogenesis and therapeutic resistance.^20^, ^21^, ^22^ Until now, the role of SphK in NEPC progression is largely unknown. It appears that SphK1 gene amplification is found in approximately 20% of NEPC and 16% of CRPC patients (Figure 1A). In contrast, Sphk2 gene amplification is not apparent in NEPC patients (Supporting information Figure S1A). RNA‐seq data from NEPC/CRPC^11^, ^26^ and NEPC sub‐lines derived from an androgen‐deprived LNCaP model further support the correlation of SphK1 elevation with NEPC (Supporting information Figure S1B). A positive correlation between the mRNA expression of SphK1 and several NETFs (such as BRN2, FOXA2 and SOX2) as well as NE markers, CgA and Syp, can be found in PCa specimens derived from TCGA database (Figure 1B). Also, IHC data from PCa TMA indicated a positive correlation between SphK1 and Syp expression (Figure 1C). Furthermore, using PCa PDE model, S1P can induce BRN2, FOXA2 and Syp protein expression (Figure 1D and Supporting information Figure S1C). Additionally, a similar correlation from two well‐characterized NEPC cell lines (such as PC3 and NCI‐H660) but not from the ADPC cell line LNCaP is observed (Supporting information Figure S1D). Indeed, elevated SphK1 protein expression was detected in a variety of cell models (such as 22RV, ARCaP‐IIB5 and ‐IIG5, and PC3) expressing NE phenotype compared with that in AR‐positive ADPC cell models (such as LNCaP, C4‐2, C4‐2B and VCAP) (Supporting information Figure S1E), however, the ubiquitous expression of SphK2 was associated with every PCa cell lines. Overall, these data support a promoting role of SphK1 in NEPC development.

To demonstrate the impact of Sphk1 on NEPC progression with androgen‐independent growth and Enzalutamide resistance, we performed constitutive active (CA) form of SphK1 with transformation at serine (S) 225 site into glutamic acid (E), which increases catalytic activity and induces translocation into the plasma membrane.^27^, ^28^ Then, LNCaP and ADPC cell lines were transfected with the constitutive active‐SphK1 (S225E) that can increase the levels of extracellular S1P (Supporting information Figure S1F) and promote androgen‐independent growth as well as the NED evidenced by the elevation of several NETFs and NE markers at both protein and mRNA levels (Figure 1E) as well as in vitro cell growth (Supporting information Figure S1F). Hence, we further investigated the role of SphK1 in developing Enzalutamide resistance of PCa by co‐culturing both vector control (VC) LNCaP labeled with mCherry and CA‐SphK1 LNCaP labeled with GFP at 100:1 ratio in the presence of Enzalutamide (Figure 1F, right panel). Long‐term (3 weeks) androgen deprivation leads to a growth advantage of CA‐SphK1 LNCaP cells. Thus, these data demonstrate that SphK1 activation is one of the key underlying mechanism of developing ADT resistance and contributes to NED in PCa cells.

### 3.2 AR in co‐operation with REST directs transcriptional repression of SphK1

**FIGURE 2:**
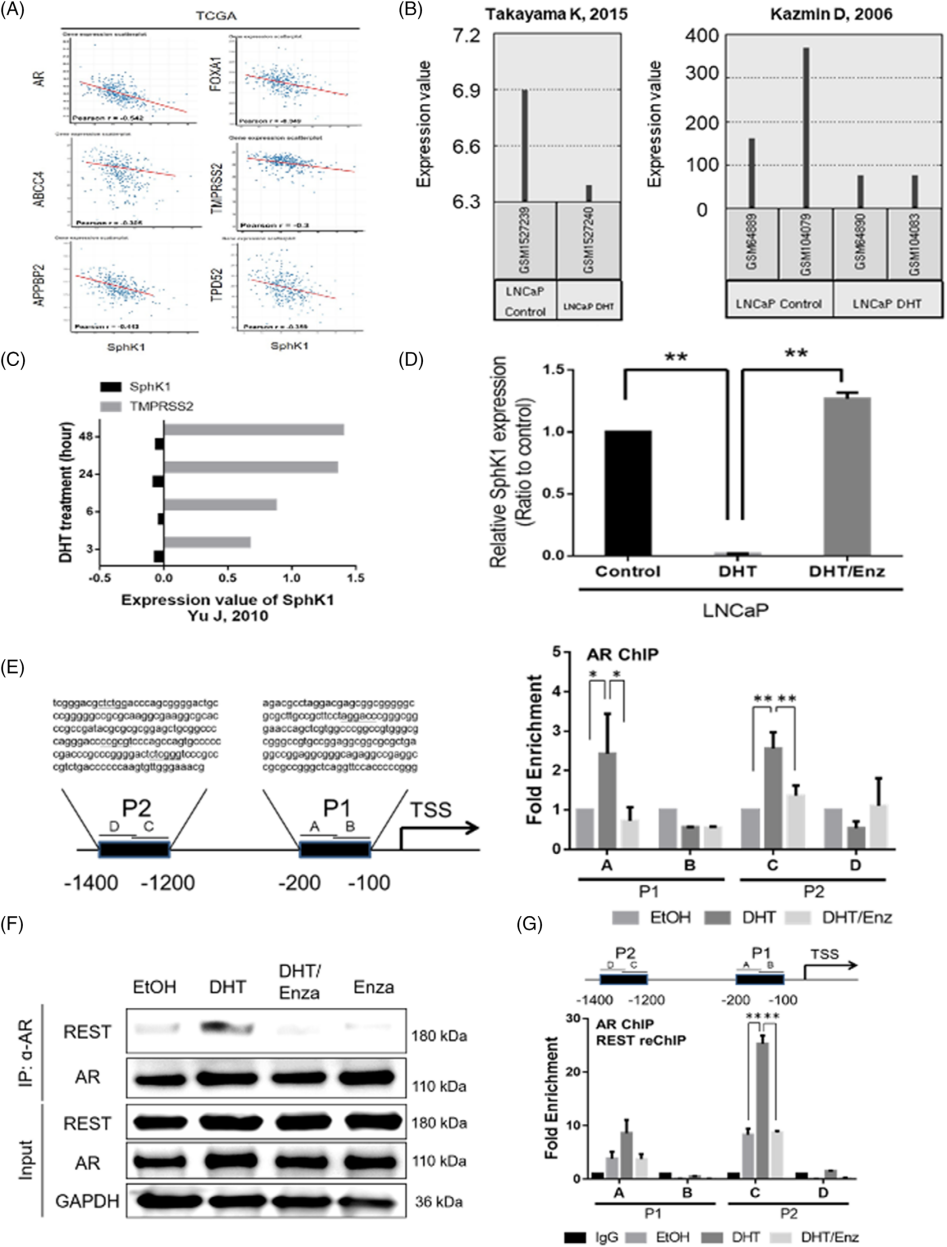
The suppressive effect of AR on Sphk1 gene expression. (A) An inverse correlation of mRNA expression between SphK1 and AR‐regulated genes (AR, ABCC4, APPBP2, TMPRSS2 and TDD52) from TCGA database. (B) Decreased SphK1 gene expression in LNCaP cells treated with vehicle or DHT for 24 h (GSE62454, GSE436). (C) Time course effect of R1881 on the expression of SphK1 and TMPRSS2 genes in LNCaP cells (GSE14097). (D) The opposite effect of 10 nM DHT or 20 μM Enzalutamide on SphK1 mRNA expression in LNCaP cells. (E) The impact of 10 nM DHT or 20 μM Enzalutamide on the binding of AR for predict androgen recognition element (ARE, underline) in SphK1 promoter at P1 (A, B) and P2 (C, D) region. (F) The impact of 10 nM DHT or 20 μM Enzalutamide on the interaction between AR and REST proteins. (G) The impact of 10 nM DHT or 20 μM Enzalutamide on the binding of AR‐REST complex to predict ARE in SphK1 promoter. *p < .05; **p < .01

Noticeably, there is an inverse correlation of AR and SphK1 protein expression in PCa (Supporting information Figures S1E and S2A), suggesting that a potential regulatory mechanism of both genes. Transcriptomic analyses of PCa databases indicated a similar negative correlation between SphK1 and AR or AR‐regulated genes (such as ABCC4, APPBP2, TMPRSS2 and TDD52)^29^ (Figure 2A and Supporting information Figure S2B). Also, GEO datasets of LNCaP cells treated with dihydrotestosterone (DHT) for 24 h (Figure 2B) or R1881 with a time course (3 to 48 h) (Figure 2C) clearly demonstrate the suppressive effect of androgen on SphK1 expression. Indeed, the inhibitory effect of DHT on SphK1 mRNA (Figure 2D) and protein (Supporting information Figure S2C) expression in LNCaP cells cultured in androgen deprived condition (i.e., phenol‐red free and charcoal‐stripped FBS) for 48 h; this inhibitory effect can be abolished in the presence of Enzalutamide (Figure 2D) or AR knockdown LNCaP cells (Left panel, Supporting information Figure S2D). This observation is further supported by the fact that Enzalutamide alone could increase SphK1 expression in LNCaP cells and Enzalutamide‐resistant LNCaP (i.e., LNCAP MDVR) cells exhibited highly elevated SphK1 (Middle panel, Supporting information Figure S2D). Consistently, increased S1P production was observed in LNCaP treated with Enzalutamide for 24 h (Right panel, Figure S2D). It is known that constitutively active AR variant (ARV7) can bind to androgen response element (ARE) motif.^30^, ^31^ Thus, ARV7 overexpression in PC3 cells can suppress SphK1 expression (Supporting information Figure S2E). Moreover, DHT can inhibit SphK1 expression in PC3 cells‐expressing AR (Supporting information Figure S2F). All these results indicate that SphK1 is an AR‐repressed gene.

We further performed an AR motif search within the region upstream of the *SPHK1* gene and found four clusters of potential ARE at the region (‐104 to ‐118, ‐1245 to ‐1259, ‐1284 to ‐1298 and ‐1373 to ‐1387) of the Sphk1 promoter. As shown in Figure 2E, two AR binding sites (A: ‐127 to ‐198 and C: ‐1215 to ‐1291) were confirmed by ChIP assay; DHT treatment increased the binding of AR to both sites, which was diminished by Enzalutamide (Figure 2E). Indeed, DHT (Figure 2F) can increase the complex formation between AR and REST known as an AR co‐repressor.^32^ In contrast, Enzalutamide can interrupt this complex formation (Figure 2F). As expected, AR ChIP‐REST reChIP data support the binding of AR‐REST complex to these two ARE sites (Figure 2G). These results support that the presence of REST is responsible for AR‐suppressed SphK1 gene transcription.

### 3.3 The enzymatic activity of Sphk1 is required for NED of PCa

**FIGURE 3:**
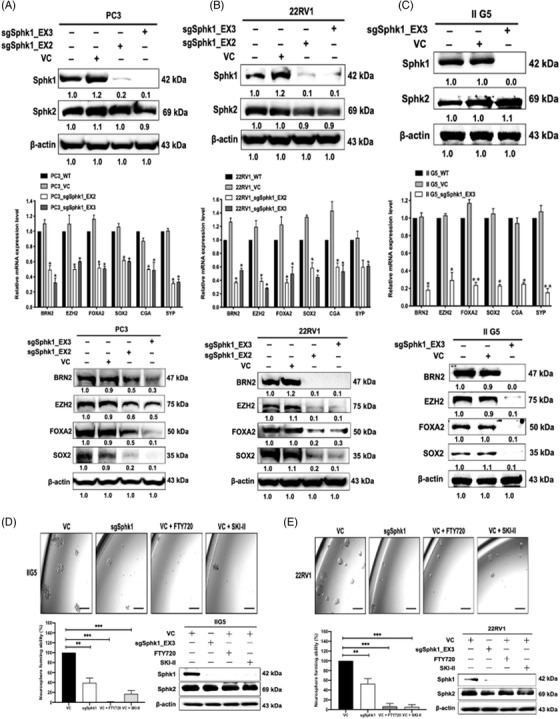
The role of SphK1 in the onset of NEPC. (A, B, C) Top panel: Decreased SphK1 protein expression in several SphK1 knockout (sgSphK1) PCa cells. Middle panel: Decreased mRNA expression of BRN2, EZH2, FOXA2, CHA and SYP gene in sgSphK1 cells. Bottom panel: Decreased protein expression of BRN2, EZH2, FOXA2 and SOX2 protein level in in sgSphK1 cells. (D, E) The effect of SphK1 on neurosphere formation of IIG5 or 22RV1 cells

To determine the role of SphK1 in the NED of PCa, the SphK1 gene was knocked out using two different sgRNA CRISPR constructs (i.e., EX2 and EX3) in several PCa cells‐expressing NE phenotypes. These constructs exhibited specific SphK1 gene knockout without changing SphK2 levels (Top panel in Figure 3A to C), which is highly associated with decreased S1P production extracellularly (Supporting information Figure S3A). Loss of SphK1 is associated with the reduced expression of NETFs (BRN2, EZH2, FOXA2 and SOX2) and NE markers (CgA and Syp) at mRNA (Middle panel in Figure 3A to C and Left panel in Supporting information Figure S3B) and protein (Bottom panel in Figure 3A to C and Right panel in Supporting information Figure S3B) levels. It is known that NE feature can be acquired through neurosphere formation as neural stem cell activities. We demonstrated that SphK1 gene knockout or enzymatic inhibitors (such as FTY720 and SKI‐II) could significantly decrease neurosphere formation in IIG5 (Figure 3D) and 22RV1 (Figure 3E) cells, indicating the driver role of SphK1 but not of Sphk2 in NED of PCa.

In Sphk1 gene knockout cells, exogenous S1P can induce NETFs (BRN2, EZH2, FOXA2 and SOX2) gene transcription in a dose‐dependent manner, where as low as serum level of S1P (1 μM) is sufficient to restore NETFs gene expression (Supporting information Figure S3C). Similar induction of NETFs and NE markers (CgA and Syp) mRNA expression by exogenous S1P was observed in LNCaP cells (Supporting information Figure S3D). Moreover, in LNCaP MDVR with a high level of SphK1, SKI‐II can decrease NETFs and NE markers mRNA (Left panel in Supporting information Figure S3E) and protein (Right panel in Supporting information Figure S3E) expression, which can be rescued by the addition of exogenous S1P. As expected, the presence of CA‐SphK1 can significantly promote neurosphere formation in ADPC cells (LNCaP and C4‐2) but SphK1 inhibitors can abolish the effect of CA‐SphK1 (Supporting information Figure S3F). These results indicate that the enzymatic activity of Sphk1 is required for the NED of PCa.

### 3.4 SphK1‐induced NED is mediated by S1P receptor (S1PR)‐MAPK pathway

**FIGURE 4:**
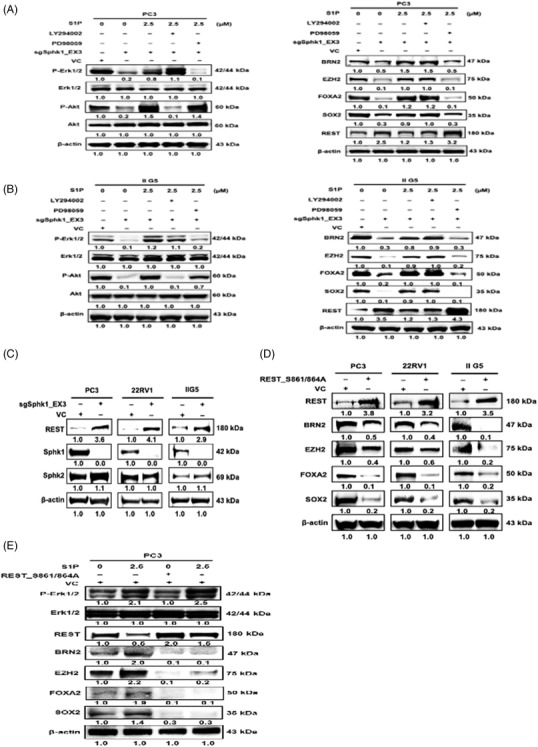
The S1P‐elicited signaling pathway leading to the onset of NEPC. (A, B) Left panel: The specificity of PI3K inhibitor (LY294002) or MEK inhibitor (PD98059) on inhibiting the activation of respective kinase (Akt S473 phosphorylation or Erk phosphorylation). Right panel: The effect of LY294002 or PD98059 on S1P‐induced NETF protein expression. (C) Accumulation of REST protein in SphK1 knockout‐ PC3, 22RV1 and IIG5 cells. (D) The effect of REST mutant (S861/864A) on NETF protein expression in PC3, 22RV1 and IIG5 cells. (E) The antagonistic effect of REST mutant (S861/864A) on S1P‐elicited NETF expression in PC3 cells. *p < .05; **p < .01

S1P action is mediated by specific S1P receptors ( S1PRs), a class of G protein‐coupled receptors that are associated with several downstream signaling pathways such as MAPK or PI3K‐Akt or JAK‐STAT.^33^ To unveil the underlying signaling pathway, SphK1 knockout PCa cells were treated with exogenous S1P before the addition of specific inhibitors for MAPK (e.g., PD98059 or GSK1120212) or PI3K‐Akt (e.g., LY294002 or BEZ235) or JAK inhibitor (e.g., Ruxolitinib) and the results (Figure 4A and B and Supporting information S4A‐C) indicated the exclusive role of MAPK pathway in S1P‐induced NED of PCa cells. In addition, S1PR1 inhibitor (Siponimod) can antagonize S1P‐induced NED of PCa cells via MAPK pathway (Supporting information Figure S4D), supporting the mechanism of action of S1P is mediated through the binding of S1PRs. Altogether, these data clearly support the key role of MAPK pathway in the autocrine induction of NED in PCa cells by SphK1.

### 3.5 MAPK‐induced REST degradation underlies SphK1‐induced NED

Knowing MAPK pathway can elicit a panel of NETFs gene expression, this action is likely mediated by a master regulator. During neurogenesis, REST is characterized as a master transcriptional repressor in silencing neuronal gene expression and is degraded in neural progenitors to promote the subsequent elaboration of a mature neuronal phenotype.^34^ In PCa, REST has been reported as the transcriptional inhibitor in IL‐6‐induced NED^35^ and hypoxia‐induced NED.^36^ In SphK1 knockout PCa cells, accumulation of REST protein is evident (Figure 4C and Supporting information Figure S4E). In contrast, S1P can reduce REST protein levels that can be reversed in the presence of MAPK inhibitor (Right panel in Figure 4A and B), implying a role of the MAPK pathway in modulating REST. A previous study^37^ demonstrated that S861/864 phosphorylation of REST facilitates the elimination of REST protein during the transition to neurons. By transfecting REST (S861/864A) construct, an unphosphorylated mutant, into several NEPC cells, a reduction of NETFs protein expression was observed (Figure 4D and Supporting information S4F). Despite elevated Erk phosphorylation in PC3 cells treated with S1P, the presence of REST (S861/864A) was able to suppress NETFs expression (Figure 4E). Also, in ADPC cells, such as C4‐2 and LNCaP, CA‐SphK1 (S225E) but not dominant‐negative (DN)‐SphK1 (S225A), can activate Erk leading to the reduction of REST, which leads to the elevation of NETFs expression (Supporting information Figure S4G). Taken together, the underlying mechanism of SphK1‐induced NED is mediated via the activation of MAPK pathway that causes the degradation of serine phosphorylated REST by Erk.

### 3.6 REST is master repressor of NETF gene transcription

**FIGURE 5:**
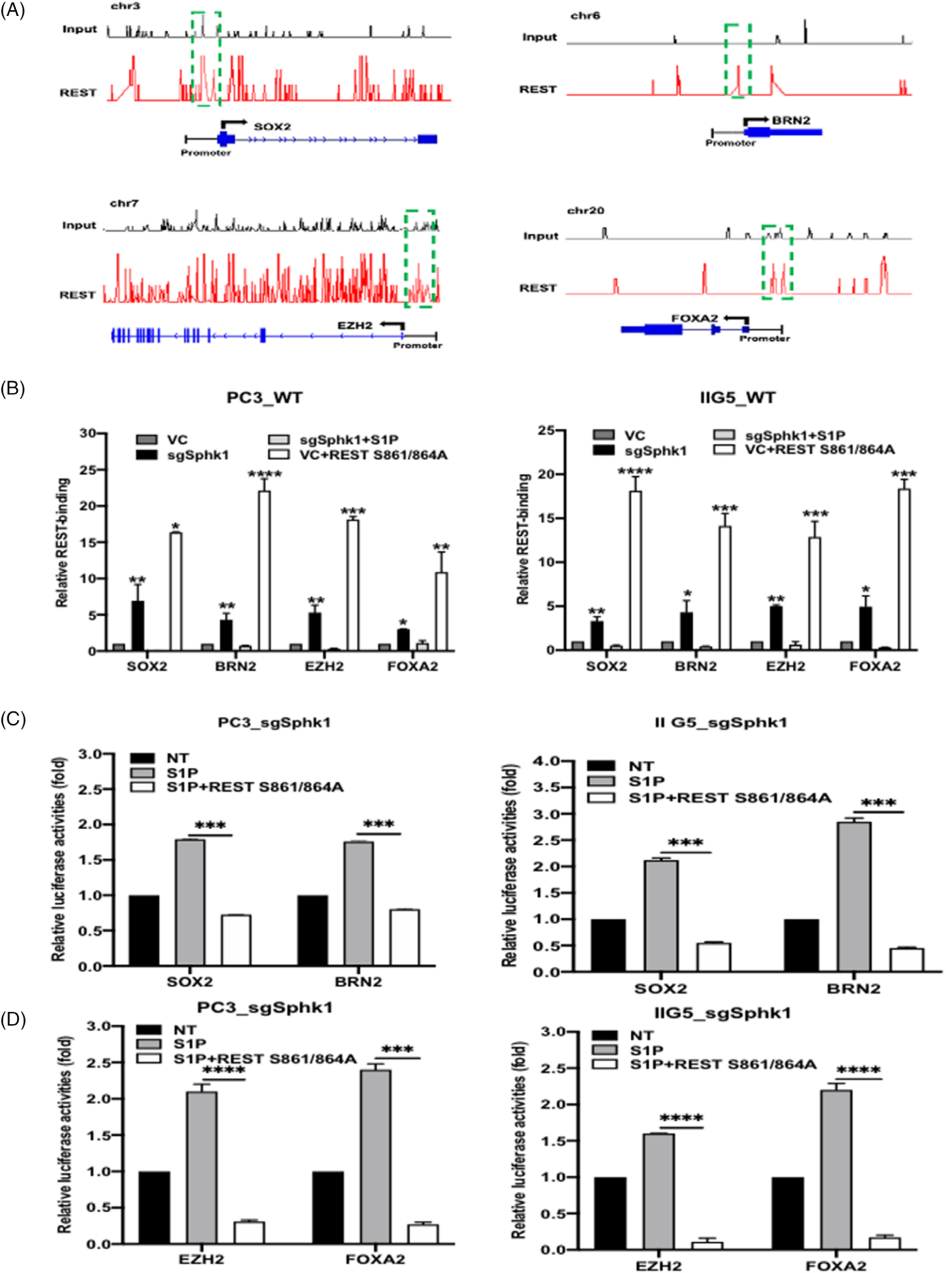
The suppressive role of REST in S1P‐induced NETF genes transcription. (A) Annotation of REST binding sites in each NETF gene promoter. (B) ChIP‐qPCR analyses of REST occupancy at promoter region of NETF genes in PC3 and IIG5 cells. (C, D) The effect of REST mutant (S861/864A) on S1P‐induced SOX2, BRN2, EZH2 and FOXA2 promoter activity in SphK1 knockout—PC3 and IIG5 cells. *p < .05; **p < .01; ***p < .001; ****p < .0001

**FIGURE 6:**
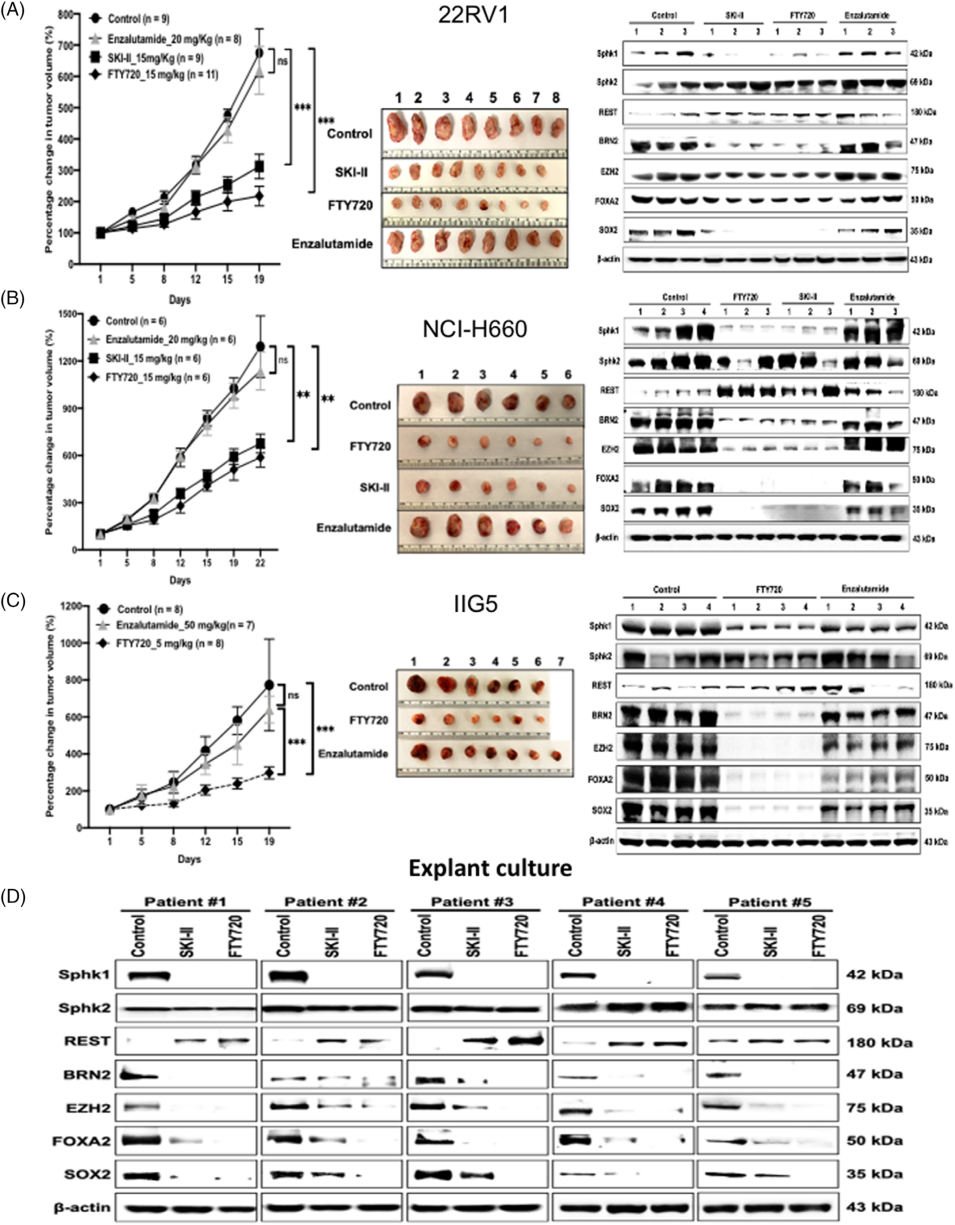
The potency of SphK1 inhibitors on NEPC therapy. (A, B, C) Left and Middle panel: The in vivo potency of SphK1 inhibitors in Enzalutamide‐resistant NEPC tumor models. Right panel: Target validation in tumor specimens harvested from the end of each treatment. (D) The inhibitory effect of SphK1 inhibitors on NETF protein expression in PDX models. **p < .01; ***p < .001

To determine the role of REST in NETF gene transcription, REST ChIP‐seq was performed to map the binding sites of REST on each NETF gene (Figure 5A). As shown in Figure 6B, Sphk1 gene knockout can increase REST binding to each NETF gene promoter, which is similar to the expression of REST (S861/864A) in either PC3 or IIG5 cell (Figure 5B). In contrast, S1P treatment can significantly decrease REST binding to each NETF gene promoter (Figure 5B). By constructing luciferase reporter vectors from each gene promoter (Supporting information Figure S5A), S1P treatment can induce the activities of each gene promoter, however, the presence of REST (S861/864A) can significantly inhibit these activities (Figure 5C and D and Supporting information Figure S5B‐E). Taken together, these results support the master suppressive role of REST in NED of PCa.

### 3.7 SphK1‐specific inhibitors are potent therapeutics for NEPC tumor

As shown in Supporting information Figure S6A, the heterogenous expression of S1PRs in each PCa cell line is noticed, suggesting that SphK1 is considered as a better druggable target. Thus, we first tested two small molecule inhibitors (SMIs) SKI‐II and FTY720 with different mechanisms of action.^33^ From a panel of 22RV1 cell models, we found that either WT or VC cell was highly sensitive to both inhibitors with IC50 approximately 5 μM (Supporting information Figure S6B). However, both SphK1 knockout cells were resistant to both inhibitors (Supporting information Figure S6A), supporting the specificity of both agents. Both inhibitors are able to reduce colony formation (Supporting information Figure S6C) and induce cell apoptosis (Supporting information Figure S6D). Also, the SphK2 inhibitor (Opaganib, ABC294640) failed to show any inhibitory effect on NEPC cells (Supporting information Figure S6E). Subsequently, we evaluated the therapeutic efficacy of SKI‐II and FTY720 (Fingolimod) in comparison with Enzalutamide using three NEPC tumor models (i.e., IIG5, 22RV1 and NCI‐H660). As expected, all three of these models are resistant to Enzalutamide (Figure 6A‐C). In contrast, both SphK1 inhibitors exhibited a significant growth inhibition (Left and Middle panel in Figure 6A‐C), which could be further validated by profiling the expression of drug target or NEPC‐associated biomarkers (Right panel in Figure 6A‐C). Noticeably, during the entire course of treatment, no significant toxicity was observed with both compounds (Supporting information Figure S6F‐H). Furthermore, by employing PDE as a pre‐clinical human model for drug target and surrogate biomarkers validation, both agents clearly reduced SphK1 and NETFs expression and exhibited target‐specificity without altering SphK2 levels (Figure 6D). In those treated specimens, we also observed elevated REST (Figure 6D), supporting the mechanism of action of SphK1 in NEPC progression. Therefore, SKI‐II and FTY720 are potent SphK1 SMIs with an immediate impact on clinical trial of NEPC therapy.

## 4 DISCUSSION

Despite initial effectiveness, second‐generation AR antagonists with higher anti‐androgen activities facilitate the progression of CRPC to less characterized NEPC with aggressive phenotypes. Until now, few therapeutic options are available due to a lack of druggable targets. Clinically, the majority of PCa is ADPC, and NEPC is rarely identified at the primary site. However, the appearance of NEPC from metastatic PCa after ADT^38^ is believed to ADPC acquired lineage plasticity and trans‐differentiation. Over the past 10 years, many efforts have been made to unveil the molecular mechanism of NEPC development. For example, the frequent mutations of TP53 and Rb1 or over‐expression of oncogenes (such as NMYC or Aurora‐Kinase A) are now recognized as genetic pre‐disposition factors. However, the mechanism‐based targeting strategy is still underdeveloped.

**FIGURE 7:**
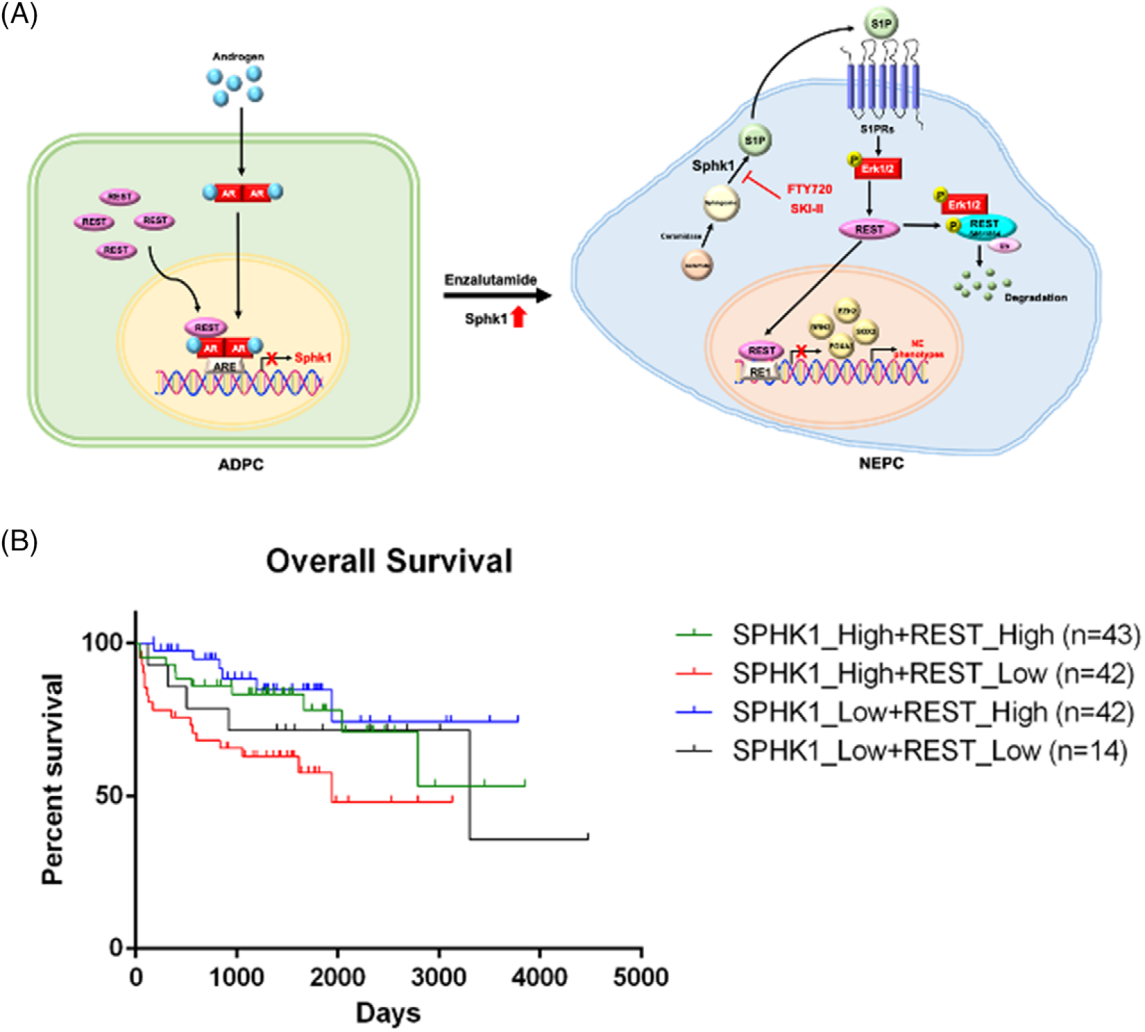
The role of SphK1 in PCa progression. (A) The scheme of the reciprocal regulator network among SphK1, AR and REST in NEPC development. (B) Clinical correlation of SphK1 and REST with overall survival of PCa patients

Based on clinical database or PCa cell models (Figure 1 and Supporting information Figure S1), SphK1 but not SphK2 is highly associated with NEPC development. Despite SphK1 and SphK2, which contribute to intracellular S1P level, but only SphK1 produces extracellular S1P (Figure 1D, Supporting information Figure S1C and F) leading to S1PR cascade in NE features. It appears that long‐term ADT leading to lost or reduced AR expression becomes apparent in clinical NEPC, implying AR may be able to suppress NEPC development. ADT is also known to generate stress on tumor‐surrounding milieu by increasing tissue hypoxia or production of many secretory factors, such as cytokines (IL‐6, IL‐8), known to induce NED of PCa,^39^ in parallel with genetic alteration. Our data conclude that SphK1 plays an autocrinal role in NEPC onset from CRPC (Figure 7A) and it is a bona fide AR‐repressed gene in PCa cells, which is regulated by the AR‐REST complex (Figure 2 and Supporting information Figure S2). REST is characterized not only as an AR co‐repressor but also as a neuron‐specific silencing factor (Figure 5) and master transcriptional repressor in neuronal cells, thus, it is often decreased in NEPC specimens.^40^

The long term of ADT is known to alter lipid metabolism,^41^, ^42^ our recent study^43^ demonstrated that peroxisome proliferator‐activated receptor γ, a major lipogenic transcription factor, is involved in IL‐6‐induced NED, indicating that the effect of lipid metabolism on NEPC development. SphK, categorized as a bioactive lipid enzyme, has two isoforms (SphK1 and SphK2) that play a central player in the sphingolipid rheostat. In mammals, SphK/S1P signaling is pivotal for normal physiology of neurogenesis, lymphocyte trafficking and vascular development through roles in cell proliferation, survival, differentiation, motility and intracellular calcium regulation.^44^, ^45^ SphK1 and SphK2 contributions to their diverse functions are due to differential expression of the number of each isozyme, conformation and dimerization properties and sub‐cellular localizations. There is a strong suggestion that imbalances of SphK1 isoform abundance may play a crucial role in the pathophysiology of diverse diseases and may contribute to resistance to current anti‐cancer drug therapies.^46^, ^47^ A wide range of S1P concentrations have been demonstrated its effect on cell proliferation or invasion from many solid tumor models^48^, ^49^, ^50^, ^51^, ^52^, ^53^, ^54^, ^55^, ^56^, ^57^, ^58^, ^59^ through binding to different S1PRs, G protein‐coupled receptors.^33^ In PCa, the role of SphK activities in cell proliferation is mediated through S1PR‐activated PI3K/Akt pathway.^60^ However, our data (Supporting information Figure S3C) from this study indicate that the serum level of S1P (∼1 μM) is sufficient to elicit NED via S1PRs (Supporting information Figure S4D). Mechanistically, our data indicate that S1P can activate MAPK pathway but not Akt or JAK‐STAT pathway in PCa (Figure 4A and B and Supporting information Figure S4A‐C) to increase REST phosphorylation at S861/864 sites leading to a rapid turnover by proteasome degradation (Figure 4C‐E and Supporting information Figure S4E‐F). Taken together, a reciprocal regulation between SphK1 and REST (Figure 7A) consistent with clinical observations supports the significant correlation between PCa with Shpk1^high^/REST^low^ with the poor overall survival (OS) of patients (Figure 7B); this pathway is independent from many genetic pre‐disposition factors found in NEPC. Similarly, the expression of SphK1 is positively correlated with poor OS and progression‐free survival (PFS) of breast cancer.^61^ Also, a strong clinical evidence^62^ indicates that lower REST expression is associated with aggressive breast cancers that most likely are estrogen receptor (ER) negative, implying the potential association of ER with REST. These parallel observations strengthen the critical role of SphK1 in PCa progression, which could prompt further investigation of the role of ER signaling in PCa progression via SphK1 activation.^63^

Our results (Figure 3D and E and Supporting information Figure S4D) indicate SphK1 or S1PRs as potential therapeutic targets. Although there are several available S1PR inhibitors, the heterogeneous expression of S1PRs in NEPC cells (data not shown) could hurdle drug selection. On the other hand, different classes of SphK1 SMIs based on its substrate structure have been developed; many of them are under clinical trial for different diseases without significant side‐effects. For example, FTY720 (Fingolimod), a S1P analog, is FDA‐approved agent for treating multiple sclerosis. In this study, we first examined the effect of FTY720, and non‐ATP‐competitive SphK1 inhibitor, SKI‐II in vitro and demonstrated that both specifically and potently targeted Sphk1 (Supporting information Figure S6A). In contrast, the SphK2 inhibitor failed to show any inhibitory effect (Supporting information Figure S6B). Furthermore, in vivo data indicated that three NEPC tumor models exhibiting Enzalutamide resistance responded both inhibitors (Figure 6A‐C). During drug administration period, animals did not show significant toxicity based on total body weight (Supporting information Figure S6C). More importantly, we demonstrate that these inhibitors can specifically reduce the expression of SphK1 (but not SphK2) and NETF proteins (Figure 6A‐C). Moreover, the drug target validation of both SphK1 inhibitors was confirmed using PDX model (Figure 6D). Our data conclude that repurposing of Sphk1 inhibitors could have an immediate impact on the development of NEPC targeted therapeutics. In addition, it is known that SphK1 conformation and activities can be influenced by several conditions including pH,^64^ guanidinium chloride^65^ and urea,^66^ new SphK1 inhibitors have been developed for breast cancer treatment,^67^ which could be applied for NEPC therapy in the future. Alternatively, SphK1 is found to interact with other kinases, such as CaMKII^68^, ^69^ that could provide additional targeting strategy for SphK1 activation in NEPC cells.

## 5 CONCLUSIONS

The appearance of NE PCa in Enzalutamide‐ or Abiraterone‐resistant CRPC patients represents lethal phenotype of PCa. Currently, there is no FDA‐approved targeted therapeutics for NEPC. This study identified clinical prevalence of a bioactive lipid kinase, SphK1, in NEPC tumors and unveiled the signaling cascade in promoting the expression of many NE regulators and factors and neuronal stem cell activities. There is a unique reciprocal regulatory network among SphK1, AR and REST in modulating NED. Based on the central role of SphK1 in NEPC development, small molecule‐specific inhibitors can overcome Enzalutamide resistance as well as tumor growth of several clinically relevant NEPC models and decrease NE biomarkers in PDE. Thus, repurposing FDA‐approved SphK1 inhibitor has an immediate translational applicability to improve the outcome of NEPC patients to prolong their OS.

## CONFLICT OF INTEREST

The authors declare that they have no conflict of interest.

## Supporting information

Supporting information
