# Supplementary material for: The central role of Sphingosine kinase 1 in the development of neuroendocrine prostate cancer (NEPC): A new targeted therapy of NEPC
Source: Clin Transl Med. 2022 Feb 20;12(2):e695. doi: 10.1002/ctm2.695 (PMC8858611; doi:10.1002/ctm2.695)
Supplement: Supplementary file 1 — Supporting information [file CTM2-12-e695-s001.pdf]

Supplementary information

Table S1. List of Primer sets

qRT-PCR	Sequences (5'- 3')
BRN2	F: ACACTGACCGATCTCCACGCAGTA
	R: GAGGGTGTGGGACCCTAAATATGAC
CgA	F: TCCGGGGTCGGGGTATATAAG
	R: CTTGGAGAGCGAGGTCTTGG
EZH2	F: GGACTCAGAAGGCAGTGGAG
	R: AAGTGTTGGGTGTTGCATGA
FOXA2	F: TGCACTCGGCTTCCAGTATG
	R: CATGTTGCTCACGGAGGAGT
GAPDH	F: CGACCTGACCTGCCGTCTAGAA
	R: GGTGTCGCTGGTGAAGTCGAGAG
S1PR1	F: CAGACAAGCAAAACAAAGTG
	R: CATCAACAAAAGTGCCAAAG
S1PR2	F: CTAGCCAGTTCTGAAAGC
	R: ACAGAGGATGACGATGAAG
S1PR3	F: GAGGAGCCCTTTTTCAAC
	R: TCATTTCAAAGGGAAGCG
S1PR4	F: GACGCTGGGTCTACTATTGCC
	R: CCTCCCGTAGGAACCACTG
S1PR5	F: AGGAAGCTCAGTTCACAG
	R: GATTCTCTAGCACGATGAAG
SOX2	F: AGCTACAGCATGATGCAGGA
	R: GGTCATGGAGTTGTACTGCA
Sphk1	F: GCTCTGGTGGTCATGTCTGG
	R: CACAGCAATAGCGTGCAGT
Syp	F: GGCTACCAGCCTGACTATGG
	R: TGGGCTTCACTGACCAGACT
**ChIP**	**Sequences (5'- 3')**
BRN2	F: GGGATGCAAGGAGAGTTGAA
	R: AGGGCTTTAAGATGGGGAAA
EZH2	F: GAGTTCGAGACCAGCCTGAC
	R: GAGTTTCGCTCTGGTTGTCC
FOXA2	F: ACCAAGAAAGAGCCTCCACA
	R: TGTCTTCCAGAGGGACTGCT
SOX2_1	F: AGCTGAGTTGGACAGGGAGA
	R: CAGGTGCCAACACTCTCTCA
SOX2_2	F: CACGACCGAAACCCTTCTTA
	R: CTCCCTGTCCAACTCAGCTC
SphK1 A (-198 to -127)	F: AGACGCCTAGGACGAGCG
	R: GGGCCACGAGCTGGTTC
SphK1 B (-35 to -161)	F: TTCCTAGGACCCGGGCG
	R: CCCGGGGGTGGAACCT
SphK1 C (-1291 to -1215)	F: CGTCCCAGCCAGTGCC
	R: CGTTTCCCAACACTTGGGGG
SphK1 D (-1389 to -1276)	F: TCGGGACGCTCTGGACC
	R: GGCACTGGCTGGGACG
**Reporter vector construction**	**Sequences (5'- 3')**
EZH2	F: GGTACCGGCAAGTGAACAGTGCTCAT
	R: CTCGAGCTCCACTGCCTTCTGAGTCC
FOXA2	F: GGTACCGCACACCTCCACGTTCACTA
	R: CTCGAGCTCTCCGACTCCTCAGACACC
**CRISPR/Cas9 gene knockout**	**Sequences (5'- 3')**
SPHK1 Ex2	F: CACCGCTGGATCCATAACCTCGACCCG
	R: AAACCGGGTCGAGGTTATGGATCCAGC
SPHK1 Ex3	F: CACCGGCGGGTTCAGCAGCACCAGCACG
	R: AAACCGTGCTGGTGCTGCTGAACCCGCC

Site-directed mutagenesis	
Sphk1 CA S225E	5'GTTCCAAGACACCTGCCGAACCCGTTGTGGTCCAGCA3'
Sphk1 DN S225A	5'GTTCCAAGACACCTGCCGCACCCGTTGTGGTCCAGCA3'

Table S2. List of antibodies

Antibody	Company	Catalog #
Actin	Santa Cruz	Sc-58673
AKT	Cell signaling	4691S
p-AKT (Ser473)	Cell signaling	4060S
AR	Sigma	A9853
BRN2	Cell signaling	12137S
CgA	Santa Cruz	Sc-393941
ERK1/2	Santa Cruz	Sc-514302
p-ERK1/2	Cell signaling	9101S
EZH2	Cell signaling	5246S
FOXA2	Abcam	Ab108422
REST	Proteintech	22242-1-AP
REST	Abclonal	A2415
ChIP Ab+TM REST	EMD Millipore	17-10456
SOX2	Cell signaling	14962S
Sphk1	Cell signaling	12071S
Sphk2	Invitrogen	PA5-51064
Synaptophysin (Syp)	Cell Signaling	5461

Figure S1. Gain of function on SphK1 associated with NEPC. (**A**) The status of SphK2 gene in different prostate cancer types (adenocarcinoma, CRPC and NEPC) from cBioPortal database. (**B**) The association of SphK1 gene expression with NEPC. (**C**) Increased NETFs (FOXA2 and BRN2) and NE markers (CgA and Syp) protein expression in PDEs treated with S1P (100 μM) 24 h. (**D**) The expression profile of SphK1, NETFs (BRN2, EZH2, FOXA2, SOX2) and NE markers (CgA, Syp) genes in LNCaP, PC3 and NCI-H660. (**E**) The expression profile of SphK1, SphK2 and AR protein in PCa cell lines. (**F**) Higher S1P production in Sphk1 CA compared with VC cells.

Figure S2. Inverse correlation between SphK1 and AR expression in PCa cell lines. (**A**) The profile of AR and SphK1 protein expression among LNCaP and its sublines. (**B**) An inverse correlation between AR (or AR-regulated genes) and SphK1 gene expression in LNCaP sublines. (**C**) The inhibitory effect of DHT on SphK1 expression in ADPC (LNCaP and C42B) cells but not in LNCaP MDVR cells. (**D**) Left panel: Elevated SphK1 expression in AR knockdown LNCaP cells. Middle panel: Elevated SphK1 expression in LNCaP treated with 20 μM Enzalutamide (EZ) for 24 h and elevated SphK1 expression in LNCaP MDVR cells with or without EZ treatment. Right panel: Increased S1P production in LNCaP treated with 20 μM EZ for 24 h. (**E**) The inhibitory effect of ARv7 on SphK1 expression in PC3 cells. (**F**) Left panel: The inhibitory effect of DHT on Sphk1 mRNA expression in AR overexpressing (OE) PC3 cells. Right panel: Elevated AR mRNA expression in PC3 cells transfected with AR expression vector.

Figure S3. The role of S1P in the onset of NEPC. (A) Decreased S1P production in SphK1 gene knockout (sgSphk1_Ex2 and _Ex3) PC3 cells. (**B**) Decreased expression of NETFs (BRN2, EZH2, FOXA2, SOX2) mRNA (Left panel) and protein (Right panel) in SphK1 knockout IIB5 cells. (**C**) Dose-dependent inductive effect of S1P on NETFs (BRN2, EZH2, FOXA2 and SOX2) gene transcription in PCa cell lines. (**D**) The inductive effect of S1P on NETFs (BRN2, EZH2, FOXA2 and SOX2) and NE markers (CgA and Syp) in LNCaP cells. (**E**) The restorative effect of S1P on SKI-II-mediated the inhibition of NETFs (BRN2, FOXA2) and NE markers (CgA and Syp) expression in LNCaP MDVR cells. (**F**) The suppressive effect of SphK1 inhibitors on neurosphere formation of LNCaP SphK1 CA or C4-2 SphK1 CA cells.

Figure S4. The mechanism of action of SphK1-elicited NETF expression. (**A**) A differential effect of MAPK inhibitor (PD98059) or Akt inhibitor (LY294002) on S1P-induced NETF mRNA expression in SphK1 knockout IIG5 cells. (**B**) A differential effect of GSK1120212 (MAPK inhibitor) or BEZ235 (PI3K inhibitor) on S1P-induced NETF protein expression in SphK1 knockout IIB5 cells. (**C**) The effect of JAK1 inhibitor (Ruxolitinib) on S1P-induced NETF protein expression in SphK1 knockout IIB5 cells. (**D**) The effect of S1PR1 inhibitor (Siponimod) on S1P-induced NETF protein expression in SphK1 knockout IIB5 cells. (**E**) Accumulation of REST protein in SphK1 knockout 22RV1 or IIB5 cells. (**F**) The inhibitory effect of REST mutant (S861/864A) on NETFs expression in IIB5 cells. (**G**) The opposite effect of CA- or DN-SphK1 on Erk activation and protein expression of REST and NETFs in C4-2 and LNCaP cells.

Figure S5. The suppressive effect of REST on NETF gene promoter activities. (**A**) The luciferase reporter construct of each NETF promoter region. (**B**) REST-ChIP data of two regions of SOX2 promoter from PC3 cells transfected with REST mutant (S861/864A) construct or SphK1 knockout PC3 cells treated with S1P. (**C**) The effect of REST mutant (S861/864A) on S1P-induced SOX2 or BRN2 promoter activity in WT or SphK1 knockout 22RV1 cells. (**D**) The effect of REST mutant (S861/864A) on S1P-induced SOX2 or BRN2 promoter activity in IIG5 and PC3 cells. (**E**) The effect of REST mutant (S861/864A) on S1P-induced EZH2 or FOXA2 promoter activity in IIG5 and PC3 cells. *** $p < 0.001$; **** $p < 0.0001$.

Figure S6. The activities of SphK1 or SphK2 inhibitors as potent targeted therapeutics. (**A**) The expression profile of S1PRs in several PCa cell lines. (**B**) The specificity of SphK1 inhibitors on the growth of Wild type (WT), Vector control (VC), SphK1 knockout 22RV1 cells. (**C**) The effect of SphK1 inhibitors on the colony formation of Wild type (WT), Vector control (VC), SphK1 knockout 22RV1 cells. (**D**) The effect of SphK1 inhibitors on the apoptosis of Wild type (WT), Vector control (VC), SphK1 knockout 22RV1 cells. (**E**) No impact of SphK2 inhibitor on in vitro cell viability of WT or SphK1 knockout 22RV1 or IIB5 cells. (**F**) No long-term toxic effect of indicated inhibitors on animals based on the loss of total body weight. (**G**) No long-term toxic toxic effect of SphK1inhibitors based on kidney and liver function. (**H**) No long-term toxic effect of SphK1 inhibitors on each organ.

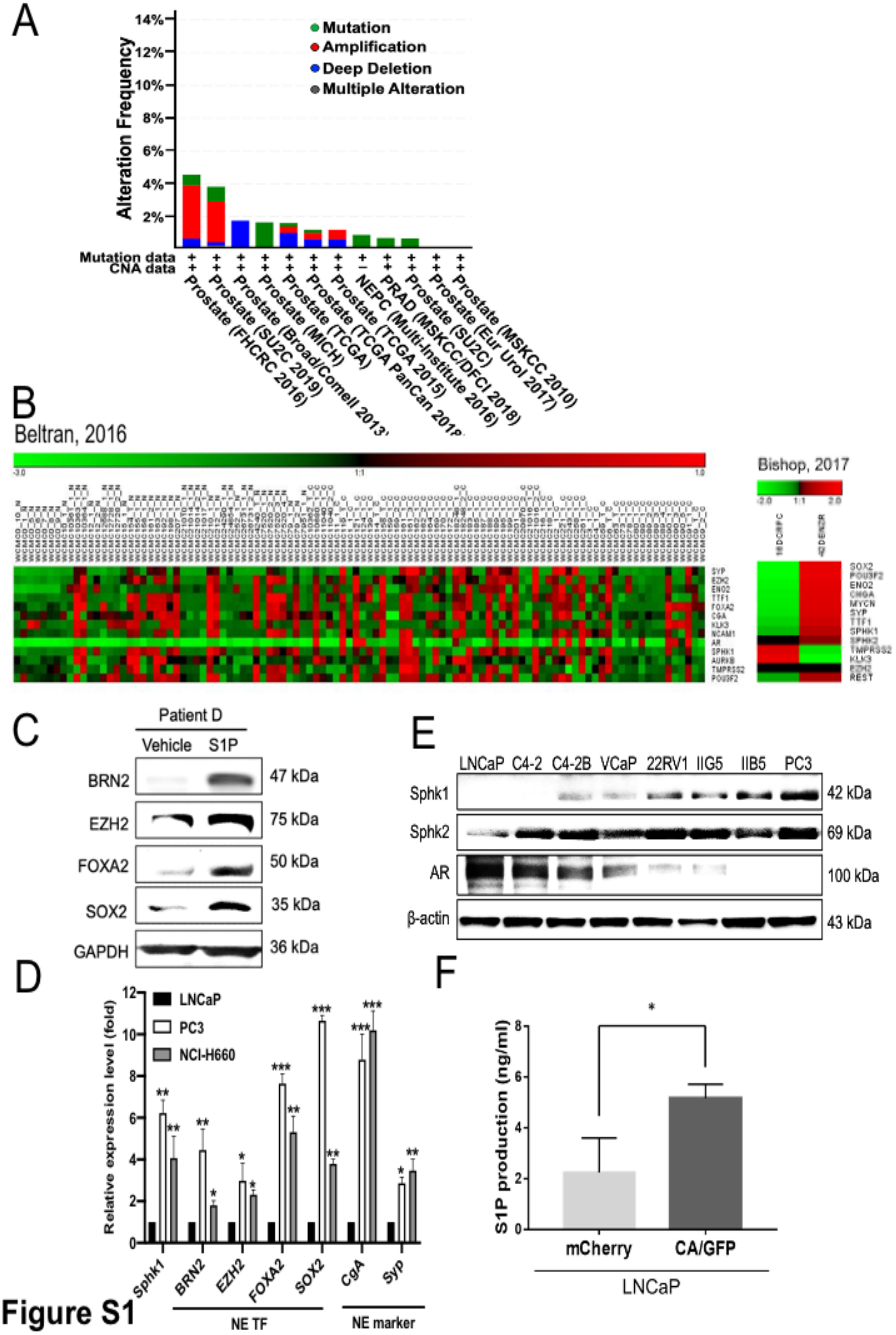
A
Mutation
Amplification
Deep Deletion
Multiple Alteration
Alteration Frequency
14%
12%
10%
8%
6%
4%
2%
Mutation data
CNA data
Prostate (FHCRC 2016)
Prostate (SU2C 2019)
Prostate (Broad/Cornell 2013)
Prostate (MICH)
Prostate (TCGA)
Prostate (TCGA PanCan 2018)
Prostate (TCGA 2015)
NEPC (Multi-Institute 2016)
PRAD (MSKCC/DFCI 2018)
Prostate (SU2C)
Prostate (Eur Urol 2017)
Prostate (MSKCC 2010)
B
Beltran, 2016
Bishop, 2017
SYP
EZH2
ENO2
TTF1
FOXA2
CGA
KLK3
NCAM1
AR
SPHK1
AURKB
TMPRSS2
POU3F2
SOX2
POU3F2
ENO2
CHGA
MYCN
SYP
TTF1
SPHK1
SPHK2
TMPRSS2
KLK3
EZH2
REST
C
Patient D
Vehicle
S1P
BRN2
EZH2
FOXA2
SOX2
GAPDH
47 kDa
75 kDa
50 kDa
35 kDa
36 kDa
E
LNCaP
C4-2
C4-2B
VCaP
22RV1
IIG5
IIB5
PC3
Sphk1
Sphk2
AR
β-actin
42 kDa
69 kDa
100 kDa
43 kDa
D
LNCaP
PC3
NCI-H660
Relative expression level (fold)
12
10
8
6
4
2
0
Sphk1
BRN2
EZH2
FOXA2
SOX2
CgA
Syp
NE TF
NE marker
F
S1P production (ng/ml)
8
6
4
2
0
mCherry
CA/GFP
LNCaP

Figure S1

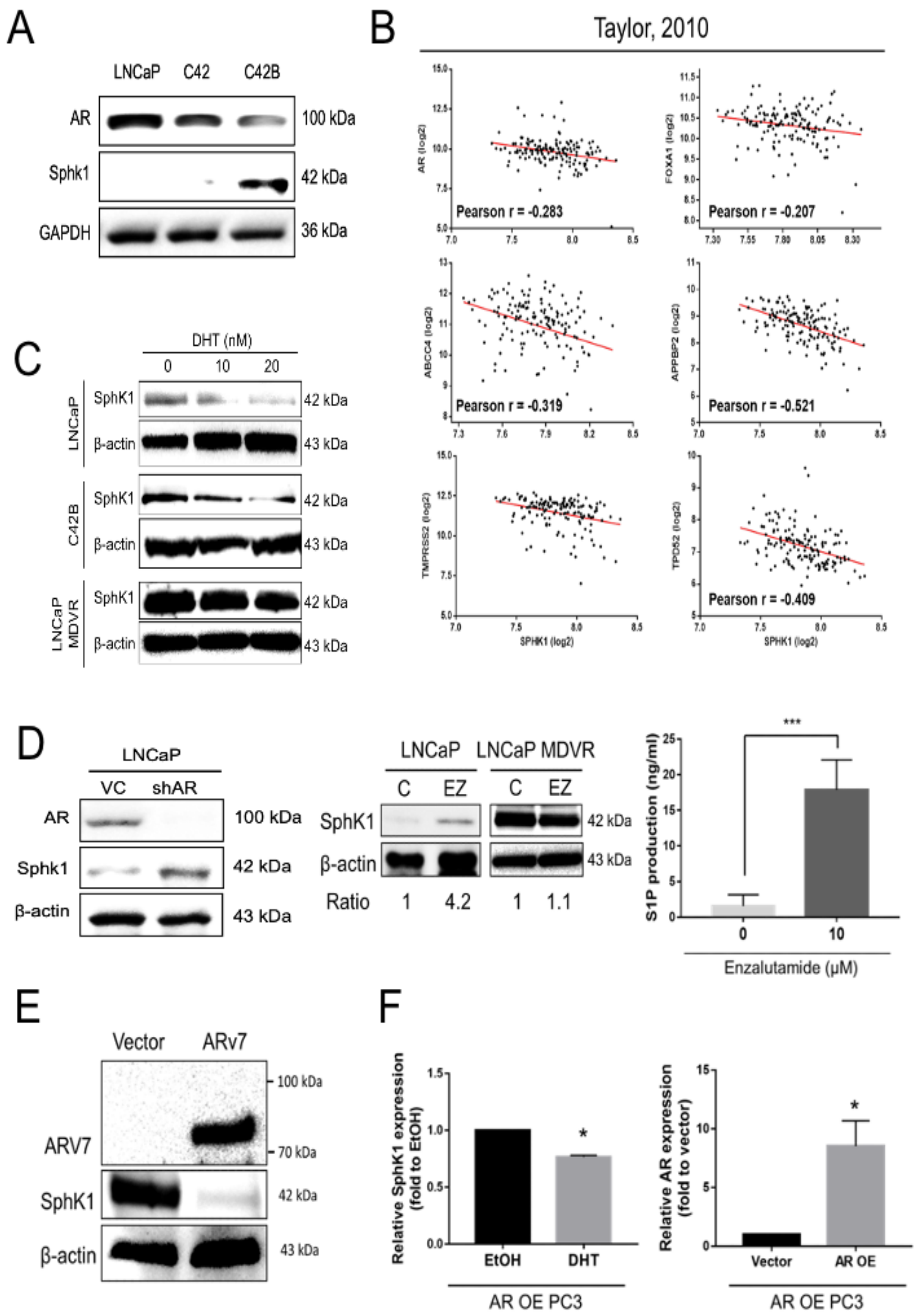
A
LNCaP
C42
C42B
AR
100 kDa
Sphk1
42 kDa
GAPDH
36 kDa
B
Taylor, 2010
Pearson r = -0.283
Pearson r = -0.207
Pearson r = -0.319
Pearson r = -0.521
Pearson r = -0.409
C
DHT (nM)
0
10
20
LNCaP
C42B
LNCaP MDVR
SphK1
β-actin
43 kDa
D
VC
shAR
LNCaP MDVR
C
EZ
Ratio
1
4.2
1
1.1
S1P production (ng/ml)
***
Enzalutamide (μM)
E
Vector
ARv7
ARV7
70 kDa
F
Relative SphK1 expression (fold to EtOH)
EtOH
DHT
AR OE PC3
Relative AR expression (fold to vector)
Vector
AR OE

Figure S2

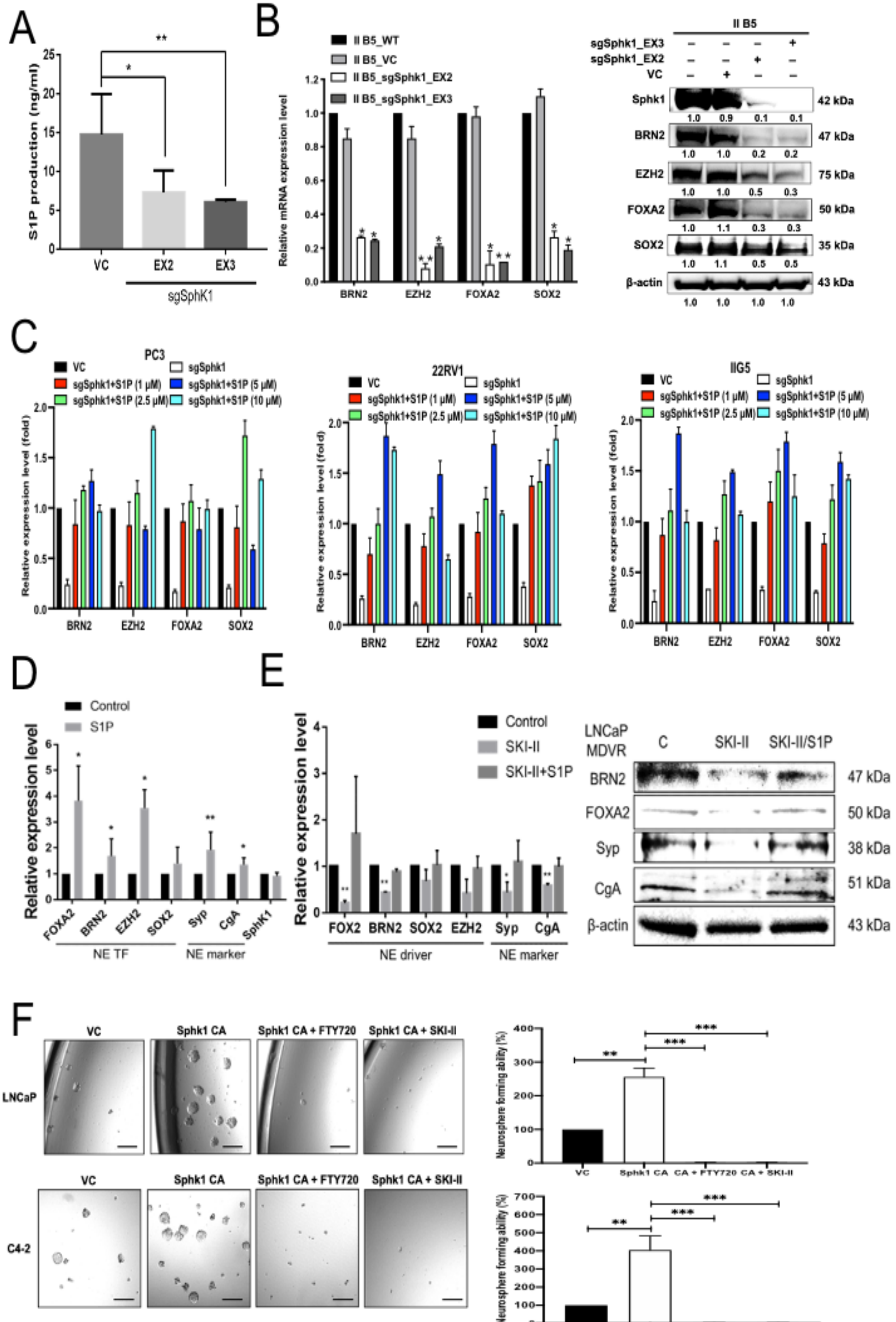

A
S1P production (ng/ml)
VC
EX2
EX3
sgSphK1
B
II B5_WT
II B5_VC
II B5_sgSphk1_EX2
II B5_sgSphk1_EX3
Relative mRNA expression level
BRN2
EZH2
FOXA2
SOX2
II B5
sgSphk1_EX3
sgSphk1_EX2
VC
Sphk1
42 kDa
BRN2
47 kDa
EZH2
75 kDa
FOXA2
50 kDa
SOX2
35 kDa
β-actin
43 kDa
C
PC3
22RV1
IIG5
VC
sgSphk1
sgSphk1+S1P (1 μM)
sgSphk1+S1P (5 μM)
sgSphk1+S1P (2.5 μM)
sgSphk1+S1P (10 μM)
Relative expression level (fold)
D
Control
S1P
Relative expression level
FOXA2
BRN2
EZH2
SOX2
Syp
CgA
SphK1
NE TF
NE marker
E
Control
SKI-II
SKI-II+S1P
Relative expression level
FOX2
BRN2
SOX2
EZH2
Syp
CgA
NE driver
NE marker
LNCaP MDVR
C
SKI-II
SKI-II/S1P
BRN2
47 kDa
FOXA2
50 kDa
Syp
38 kDa
CgA
51 kDa
β-actin
43 kDa
F
VC
Sphk1 CA
Sphk1 CA + FTY720
Sphk1 CA + SKI-II
LNCaP
C4-2
Neurosphere forming ability (%)
VC
Sphk1 CA
CA + FTY720
CA + SKI-II

Figure S3

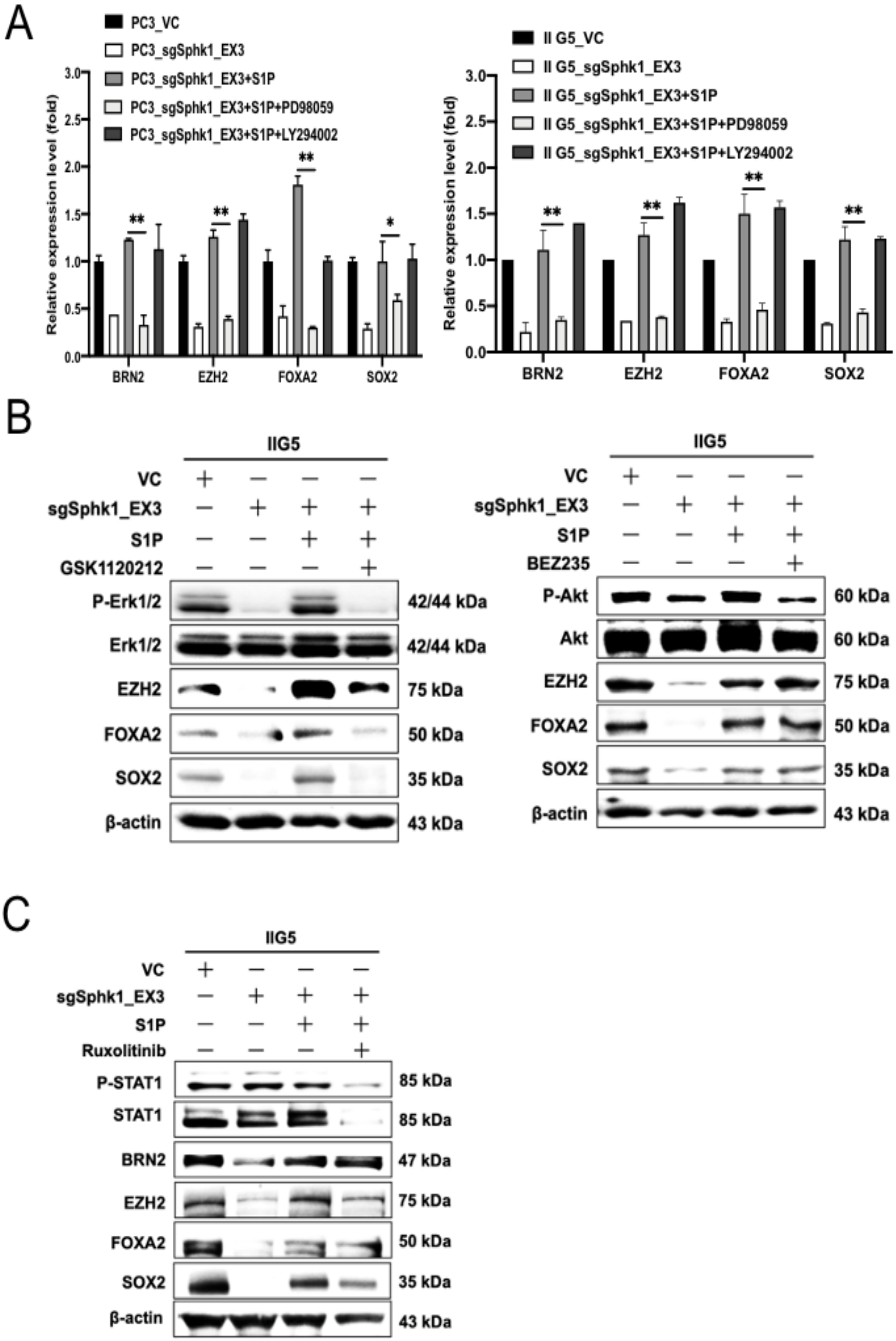
A
PC3_VC
PC3_sgSphk1_EX3
PC3_sgSphk1_EX3+S1P
PC3_sgSphk1_EX3+S1P+PD98059
PC3_sgSphk1_EX3+S1P+LY294002
Relative expression level (fold)
BRN2
EZH2
FOXA2
SOX2
II G5_VC
II G5_sgSphk1_EX3
II G5_sgSphk1_EX3+S1P
II G5_sgSphk1_EX3+S1P+PD98059
II G5_sgSphk1_EX3+S1P+LY294002
B
IIG5
VC
sgSphk1_EX3
S1P
GSK1120212
P-Erk1/2
Erk1/2
EZH2
FOXA2
SOX2
β-actin
42/44 kDa
75 kDa
50 kDa
35 kDa
43 kDa
BEZ235
P-Akt
Akt
60 kDa
C
Ruxolitinib
P-STAT1
STAT1
BRN2
85 kDa
47 kDa

Figure S4

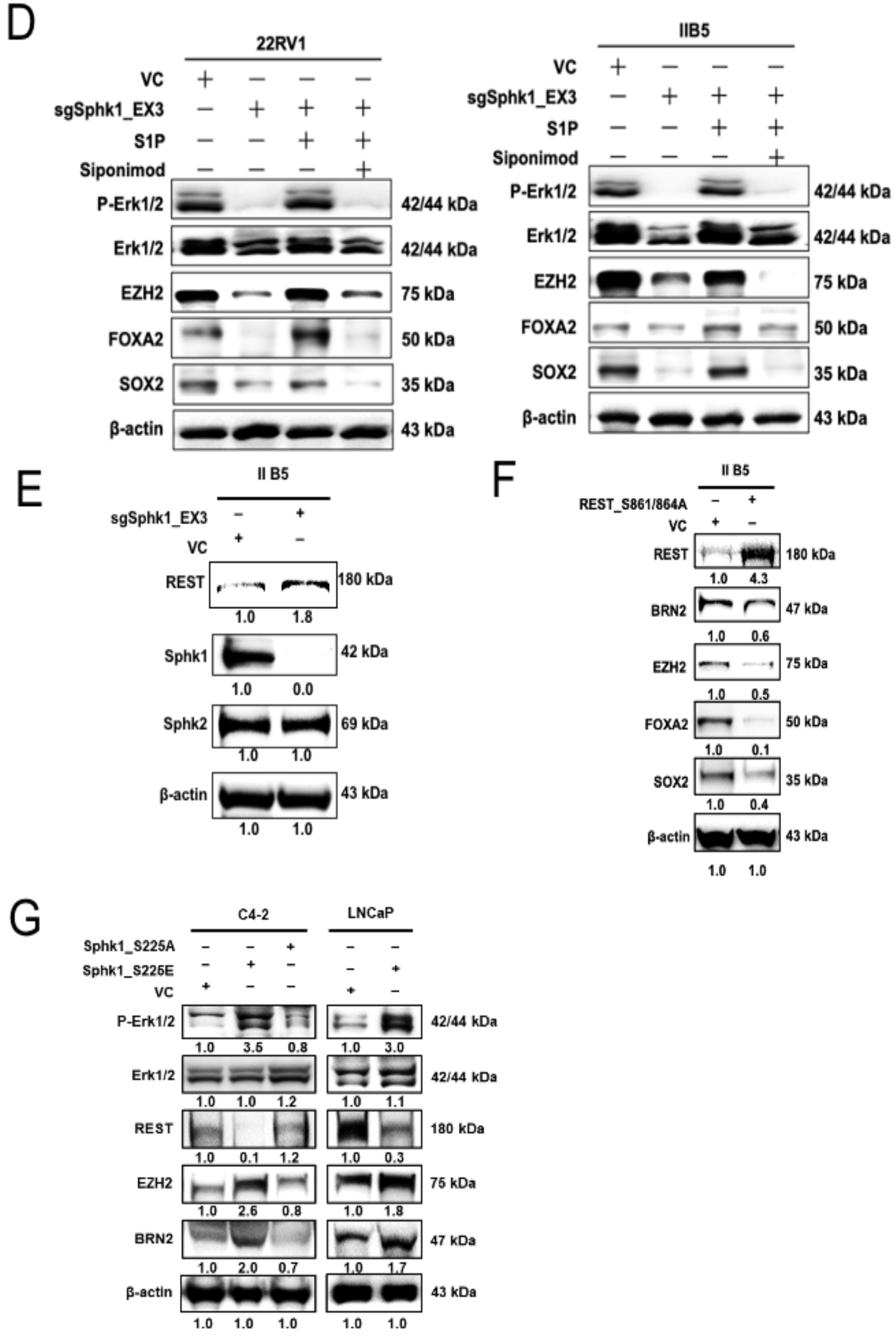
D
22RV1
VC
sgSphk1_EX3
S1P
Siponimod
P-Erk1/2
Erk1/2
EZH2
FOXA2
SOX2
β-actin
42/44 kDa
42/44 kDa
75 kDa
50 kDa
35 kDa
43 kDa
IIB5
VC
sgSphk1_EX3
S1P
Siponimod
P-Erk1/2
Erk1/2
EZH2
FOXA2
SOX2
β-actin
42/44 kDa
42/44 kDa
75 kDa
50 kDa
35 kDa
43 kDa
E
II B5
sgSphk1_EX3
VC
REST
180 kDa
1.0 1.8
Sphk1
42 kDa
1.0 0.0
Sphk2
69 kDa
1.0 1.0
β-actin
43 kDa
1.0 1.0
F
II B5
REST_S861/864A
VC
REST
180 kDa
1.0 4.3
BRN2
47 kDa
1.0 0.6
EZH2
75 kDa
1.0 0.5
FOXA2
50 kDa
1.0 0.1
SOX2
35 kDa
1.0 0.4
β-actin
43 kDa
1.0 1.0
G
C4-2
LNCaP
Sphk1_S225A
Sphk1_S225E
VC
P-Erk1/2
42/44 kDa
1.0 3.6 0.8 1.0 3.0
Erk1/2
42/44 kDa
1.0 1.0 1.2 1.0 1.1
REST
180 kDa
1.0 0.1 1.2 1.0 0.3
EZH2
75 kDa
1.0 2.6 0.8 1.0 1.8
BRN2
47 kDa
1.0 2.0 0.7 1.0 1.7
β-actin
43 kDa
1.0 1.0 1.0 1.0 1.0

Figure S4

A

B

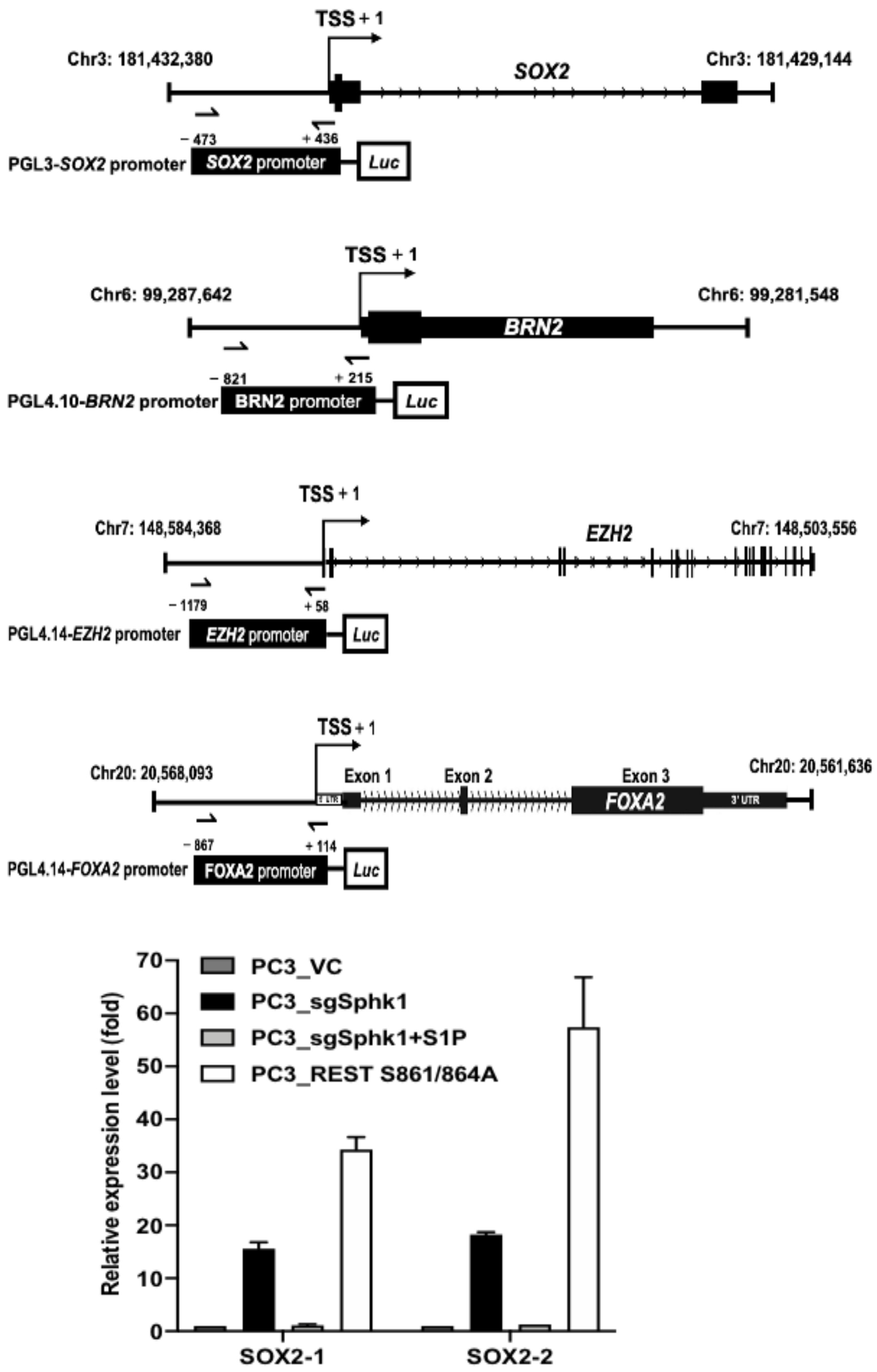
TSS + 1
Chr3: 181,432,380
SOX2
Chr3: 181,429,144
− 473
+ 436
PGL3-SOX2 promoter
SOX2 promoter
Luc
TSS + 1
Chr6: 99,287,642
BRN2
Chr6: 99,281,548
− 821
+ 215
PGL4.10-BRN2 promoter
BRN2 promoter
Luc
TSS + 1
Chr7: 148,584,368
EZH2
Chr7: 148,503,556
− 1179
+ 58
PGL4.14-EZH2 promoter
EZH2 promoter
Luc
TSS + 1
Chr20: 20,568,093
Exon 1
Exon 2
Exon 3
FOXA2
3' UTR
Chr20: 20,561,636
− 867
+ 114
PGL4.14-FOXA2 promoter
FOXA2 promoter
Luc
PC3_VC
PC3_sgSphk1
PC3_sgSphk1+S1P
PC3_REST S861/864A
Relative expression level (fold)
0
10
20
30
40
50
60
70
SOX2-1
SOX2-2

Figure S5

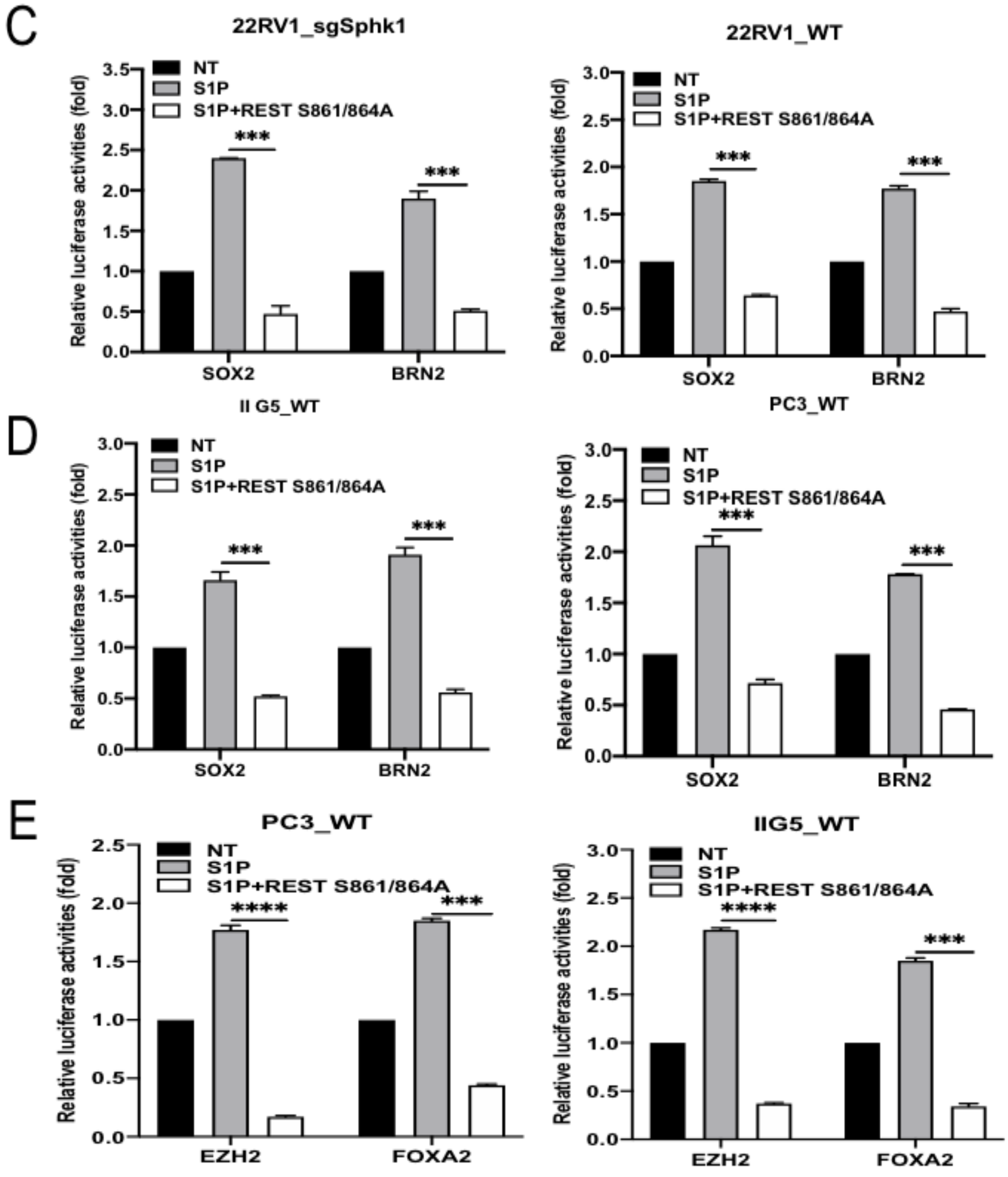
C
22RV1_sgSphk1
22RV1_WT
NT
S1P
S1P+REST S861/864A
Relative luciferase activities (fold)
***
SOX2
BRN2
D
II G5_WT
PC3_WT
E
PC3_WT
IIG5_WT
****
EZH2
FOXA2

Figure S5

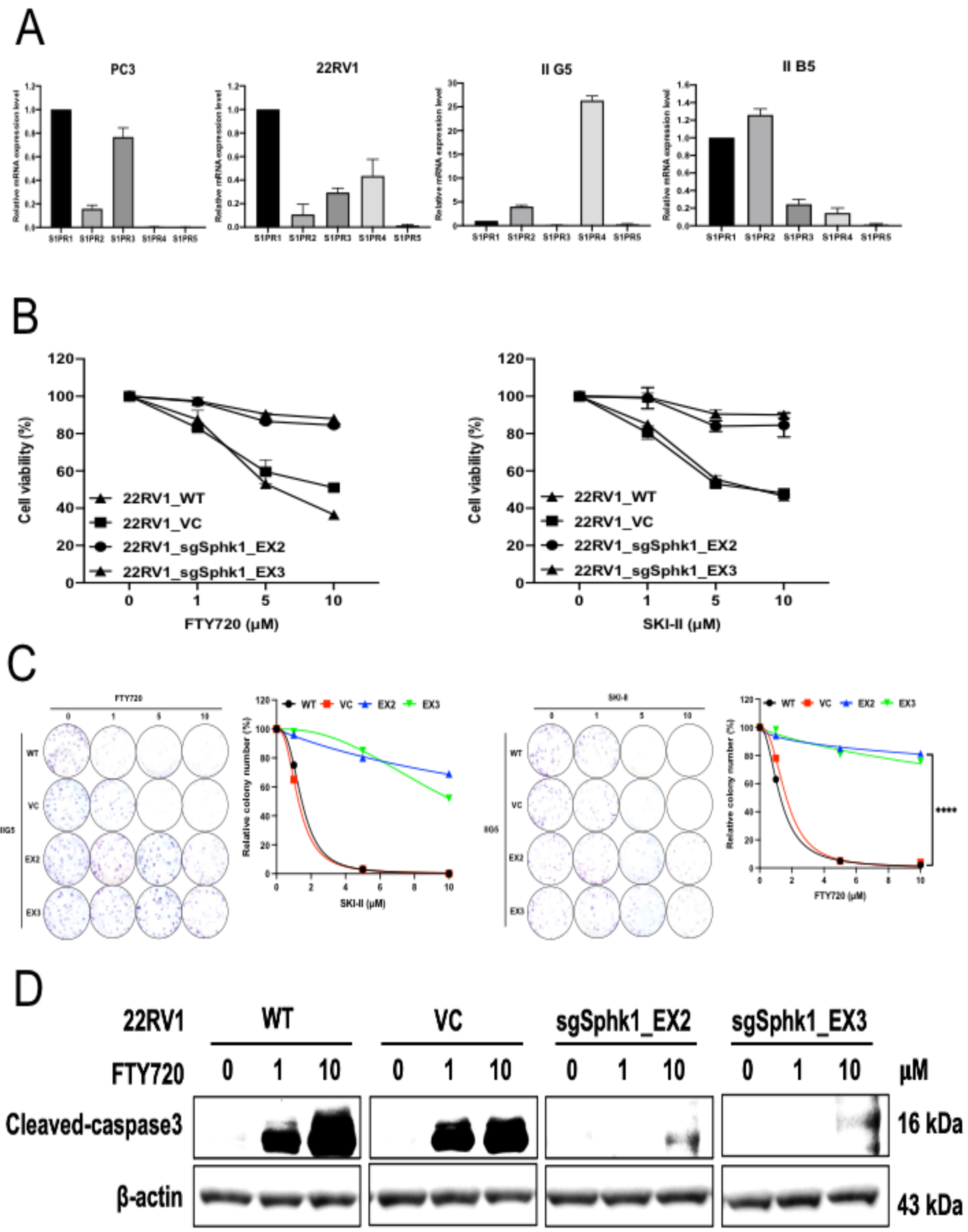
A
PC3
22RV1
II G5
II B5
Relative mRNA expression level
S1PR1
S1PR2
S1PR3
S1PR4
S1PR5
B
Cell viability (%)
22RV1_WT
22RV1_VC
22RV1_sgSphk1_EX2
22RV1_sgSphk1_EX3
FTY720 (μM)
SKI-II (μM)
C
FTY720
SKI-II
IIG5
WT
VC
EX2
EX3
Relative colony number (%)
SKI-II (μM)
FTY720 (μM)
****
D
22RV1
WT
VC
sgSphk1_EX2
sgSphk1_EX3
FTY720
0
1
10
μM
Cleaved-caspase3
β-actin
16 kDa
43 kDa

Figure S6

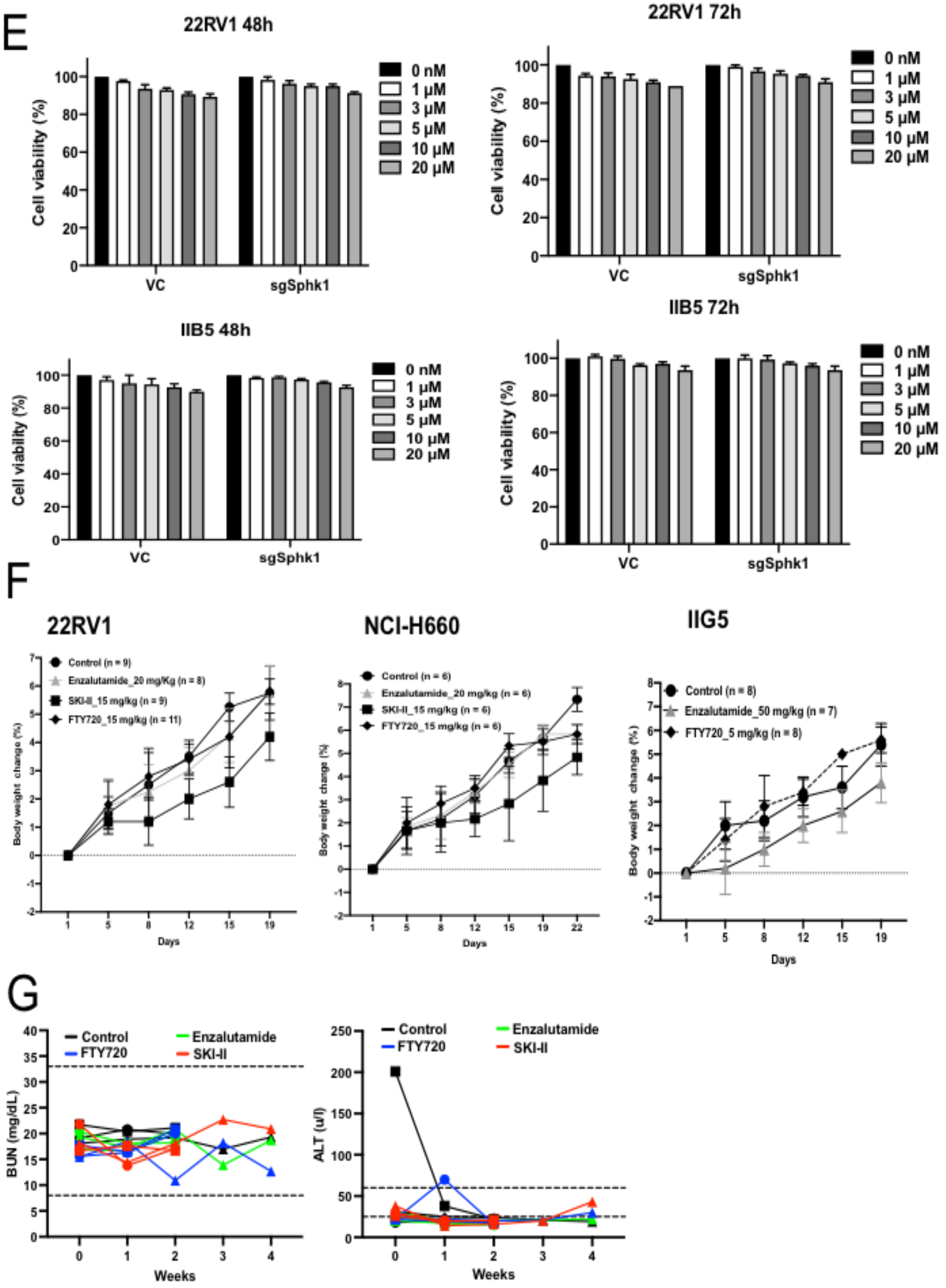
E
22RV1 48h
22RV1 72h
IIB5 48h
IIB5 72h
Cell viability (%)
0 nM
1 μM
3 μM
5 μM
10 μM
20 μM
VC
sgSphk1
F
22RV1
NCI-H660
IIG5
Control (n = 9)
Enzalutamide_20 mg/Kg (n = 8)
SKI-II_15 mg/kg (n = 9)
FTY720_15 mg/kg (n = 11)
Control (n = 6)
Enzalutamide_20 mg/kg (n = 6)
SKI-II_15 mg/kg (n = 6)
FTY720_15 mg/kg (n = 6)
Control (n = 8)
Enzalutamide_50 mg/kg (n = 7)
FTY720_5 mg/kg (n = 8)
Body weight change (%)
Days
G
Control
Enzalutamide
FTY720
SKI-II
BUN (mg/dL)
ALT (u/l)
Weeks

Figure S6

H

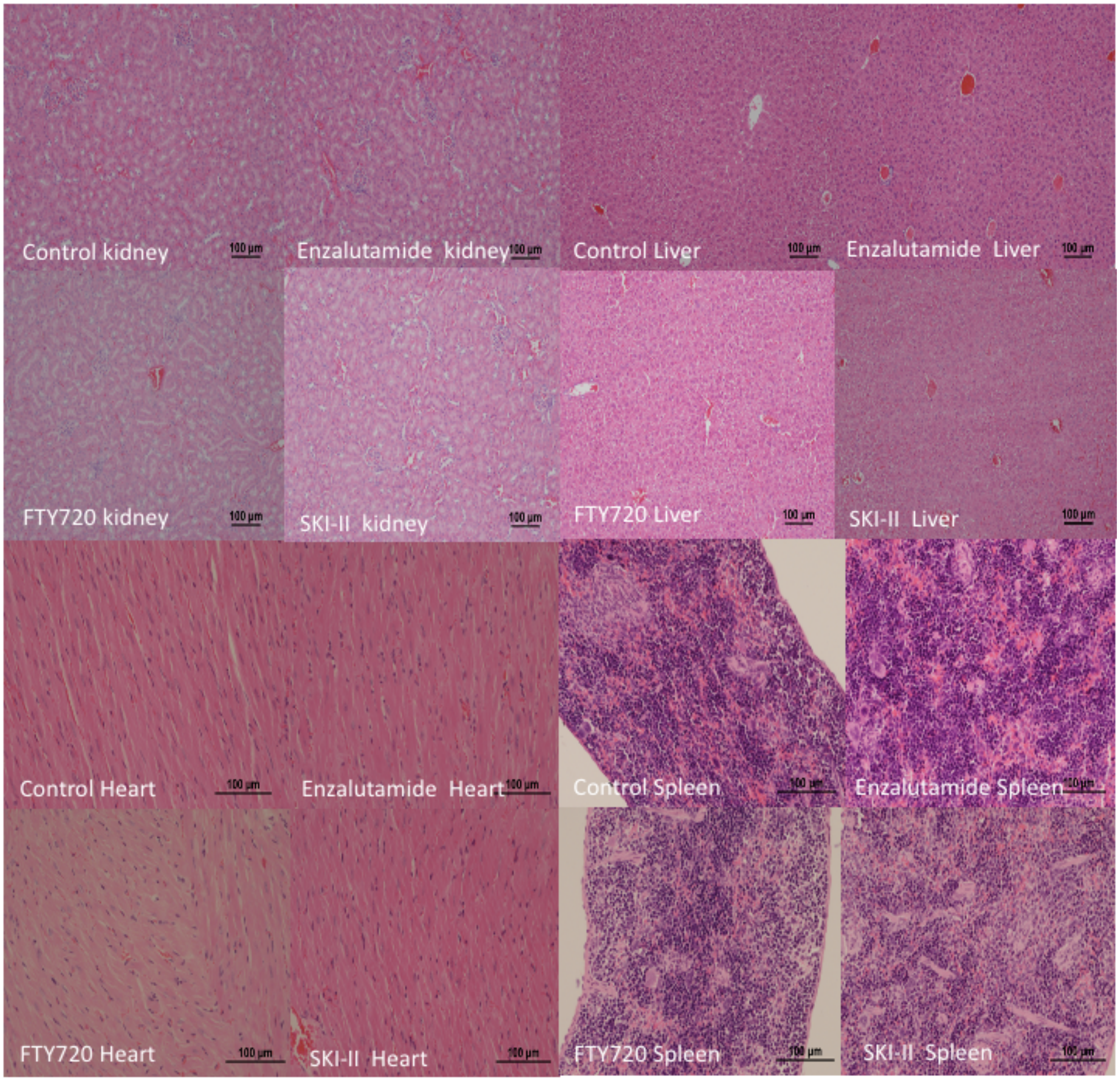

Control kidney
100 µm
Enzalutamide kidney
100 µm
Control Liver
100 µm
Enzalutamide Liver
100 µm
FTY720 kidney
100 µm
SKI-II kidney
100 µm
FTY720 Liver
100 µm
SKI-II Liver
100 µm
Control Heart
100 µm
Enzalutamide Heart
100 µm
Control Spleen
Enzalutamide Spleen
FTY720 Heart
100 µm
SKI-II Heart
100 µm
FTY720 Spleen
SKI-II Spleen

Figure S6
